# Mercury contamination is an invisible threat to declining migratory shorebirds along the East Asian-Australasian Flyway

**DOI:** 10.1038/s42003-024-06254-x

**Published:** 2024-05-16

**Authors:** Yanju Ma, Chi-Yeung Choi, Lihai Shang, Marcel Klaassen, Zhijun Ma, Qing Chang, Veerle L. B. Jaspers, Qingquan Bai, Tao He, Katherine K-S. Leung, Chris J. Hassell, Roz Jessop, Luke Gibson

**Affiliations:** 1 School of Environmental Science and Engineering, Southern University of Science and Technology, Shenzhen, China; 2 Key Laboratory of Ecology of Rare and Endangered Species and Environmental Protection (Guangxi Normal University), Ministry of Education, Guilin, 541006 Guangxi China; 3 Guangxi Key Laboratory of Rare and Endangered Animal Ecology, College of Life Sciences, Guangxi Normal University, Guilin, 541006 Guangxi China; 4 Environmental Research Center, Duke Kunshan University, Kunshan, 215316 Jiangsu China; 5 State Key Laboratory of Environmental Geochemistry, Institute of Geochemistry, Chinese Academy of Sciences, Guiyang, 550081 Guizhou China; 6 School of Life and Environmental Sciences, Deakin University, Geelong, VIC Australia; 7 Ministry of Education Key Laboratory for Biodiversity Science and Ecological Engineering, Coastal Ecosystems Research Station of the Yangtze River Estuary, Institute of Biodiversity Science, School of Life Sciences, Fudan University, Shanghai, 200433 China; 8 Nanjing Normal University, Nanjing, 210024 Jiangsu China; 9 Department of Biology, Norwegian University of Science and Technology (NTNU), 7491 Trondheim, Norway; 10 Dandong Forestry and Grassland Development Service Center, Dandong, 118000 Liaoning China; 11 Zhanjiang Mangrove National Nature Reserve Bureau, Zhangjiang, Guangdong, 524000 China; 12 Hong Kong Waterbirds Ringing Group, Mai Po Nature Reserve, Mai Po, Hong Kong, China; 13 Australian Wader Studies Group, Curtin, ACT 2605 Australia; 14 Victorian Wader Study Group, Thornbury, VIC 3071 Australia

**Keywords:** Animal migration, Conservation biology, Biodiversity

## Abstract

Exposure to pollutants is a potentially crucial but overlooked driver of population declines in shorebirds along the East Asian-Australasian Flyway. We combined knowledge of moult strategy and life history with a standardised sampling protocol to assess mercury (Hg) contamination in 984 individuals across 33 migratory shorebird species on an intercontinental scale. Over one-third of the samples exceeded toxicity benchmarks. Feather Hg was best explained by moulting region, while habitat preference (coastal obligate vs. non-coastal obligate), the proportion of invertebrates in the diet and foraging stratum (foraging mostly on the surface vs. at depth) also contributed, but were less pronounced. Feather Hg was substantially higher in South China (Mai Po and Leizhou), Australia and the Yellow Sea than in temperate and Arctic breeding ranges. Non-coastal obligate species (*Tringa* genus) frequently encountered in freshwater habitats were at the highest risk. It is important to continue and expand biomonitoring research to assess how other pollutants might impact shorebirds.

## Abstract (web summary)

Over one-third of the sampled shorebirds along the East Asian-Australasian Flyway are facing Hg risk. *Tringa* genus in South China was at the highest risk. Feather Hg was best explained by feathers’ moulting region, while habitat preference, diet, and foraging stratum were less important.

## Introduction

The East Asian-Australasian Flyway (EAAF) is one of the world’s most important migratory flyways, stretching from the Russian Arctic and Alaska to southern Australia and New Zealand^1,2^. It supports approximately 8 million migratory shorebirds (order Charadriiformes) moving annually at a continental scale^3^. Unfortunately, many of these species have recently experienced steep population declines^4^ and over 20 species are now classified as globally threatened by the IUCN Red List^5^. Previous studies have recognised climate change and habitat loss or modification as major contributing factors in shorebird declines^4,6,7^. Additionally, shorebirds could be vulnerable to environmental contamination^8–10^ since they rely heavily on wetland habitats. Anthropogenic activities regularly subject these habitats to residues of trace elements and emerging and persistent organic chemicals^11–13^. Even at relatively pristine breeding sites in remote northern latitudes, volatile and semi-volatile pollutants have been detected in high concentrations via long-range atmospheric transportation^14^, including in Alaska^15^ and the Canadian Arctic^16^.

Among pollutants, mercury (Hg) and its most toxic form methylmercury are of particular concern in birds^17^. These contaminants have detrimental impacts on long-distance migrants via impaired takeoff^18^, affected migratory behaviour^19^, reduced endurance flight performance^20^, altered stopover decisions^21^, and eventually reduced survival^22^. Polychaeta, a key prey for many shorebird species, is a common vector for methylmercury bioaccumulation in shorebirds^23^. Shorebirds are thought to be highly exposed to methylmercury since they generally forage in wetlands, which are known to be contaminated by Hg via atmospheric deposition and runoff ^24–26^. Furthermore, several shorebird species (e.g., Bar-tailed Godwit *Limosa lapponica*) along the EAAF are among the champions of the longest wildlife migrations worldwide^27^. Their migration is physically and physiologically demanding^28^, and thus, additional stress such as environmental contamination could have fatal consequences.

Distinguishing environmental Hg exposure and potential risks to migratory birds spending their lives on a vast spatial scale is crucial for their conservation^10^. Large-scale assessment of environmental Hg accumulation in shorebirds can help identify the distribution of contamination loads in various migrant species, and also provide guidelines for effective conservation strategy. Limited research for breeding shorebirds has been conducted in Alaska, northern Canada^15,29,30^, and Greenland^31^. Studies on shorebirds during the non-breeding stage have been restricted to only a few common species (e.g., Kentish Plover *Charadrius alexandrinus*^32,33^), and key stopover sites such as Delaware Bay in North America^34^ and Pertuis Charentais in France^35,36^. The Asia-Pacific region contributes the largest proportion (~49%) of global Hg emissions^37^, which overlaps with sites of many migratory shorebirds along the EAAF^38^. Although recent studies have investigated Hg exposure at a staging site in Taiwan and in the non-breeding ground of Western Australia^39^, there is a paucity of information about Hg exposure and its ecological risks to shorebirds travelling along the full length of the EAAF, the most threatened but least studied flyway^10^.

In the avian ecotoxicological field, feathers have been widely used for contaminant biomonitoring because they involve a non-lethal sampling technique and are easily preserved^40–44^. During avian moulting, the growth of feathers sequesters circulating Hg, making feathers an important indicator of Hg bioaccumulation^45^. Although there has been some controversy related to the variability of Hg concentration in different feathers at different times^46^, recent studies have confirmed the feasibility of using feathers in Hg monitoring with careful consideration of the specific moulting patterns amongst bird species, populations and age classes^47–49^. Specifically, Bottini et al.^47^ showed the strong correlation (*R*^2^ > 0.90) between Hg concentration in blood and feathers, opening the way to use flight feathers (primary, secondary, tail, and covert feathers) for which moult patterns have been carefully documented in species of interest to evaluate Hg burden at the times and sites of feather growth. For example, Ma et al. ^50^ applied this approach to establish Hg exposure for North American passerines across their breeding grounds, covering a wide geographic range. Moreover, only small feather samples are required to measure Hg exposure accurately^49^.

Here, we investigated Hg exposure in migratory shorebirds along the EAAF by combining knowledge of species-specific moult patterns and standardised sampling of feathers for Hg measurement. We aimed to 1) assess differences in Hg contamination across different shorebird species in feathers grown at different locations during their life cycle (breeding vs. non-breeding areas); 2) estimate the ecological risks of Hg to migratory shorebird species along the EAAF using multiple risk thresholds; and 3) explain variation in Hg exposure as a function of region, habitat preference, diet, and foraging strategy.

## Results

### Patterns in Hg accumulation depending on the location of feather growth

Overall, we measured feather (the 6th primary covert) total mercury (hereafter THg) concentrations (unit: mg/kg, dry weight) from 984 individuals across 33 species, representing Hg exposures ranging from the Arctic breeding grounds to the Australian non-breeding grounds, covering almost the complete latitudinal span of the EAAF. The average THg concentration for all samples was 1.87 ± 2.27 mg/kg (median: 1.28 mg/kg), ranging from 0.11 to 38.63 mg/kg. Without considering species and site variations and ignoring data with unidentified origin (*n* = 108), feathers from non-breeding grounds (stopovers and wintering grounds, indicated by feathers from 1 juvenile Curlew Sandpiper *Calidris ferruginea* with active moult and 427 adults) had a THg concentration of 2.09 ± 2.74 mg/kg (median: 1.38 mg/kg, range: 0.25–38.63 mg/kg, *n* = 428). This was significantly higher (nearly 1.4 times, *F*_1, 869.16_ = 50.16, *p* < 0.0001) than THg concentration in feathers from the breeding grounds (1.51 ± 1.76 mg/kg; median: 0.99 mg/kg, range: 0.11–17.83 mg/kg, *n* = 448; using feathers from juveniles).

### Species variations in feather THg concentrations and ecological risks along the EAAF

**Fig. 1:**
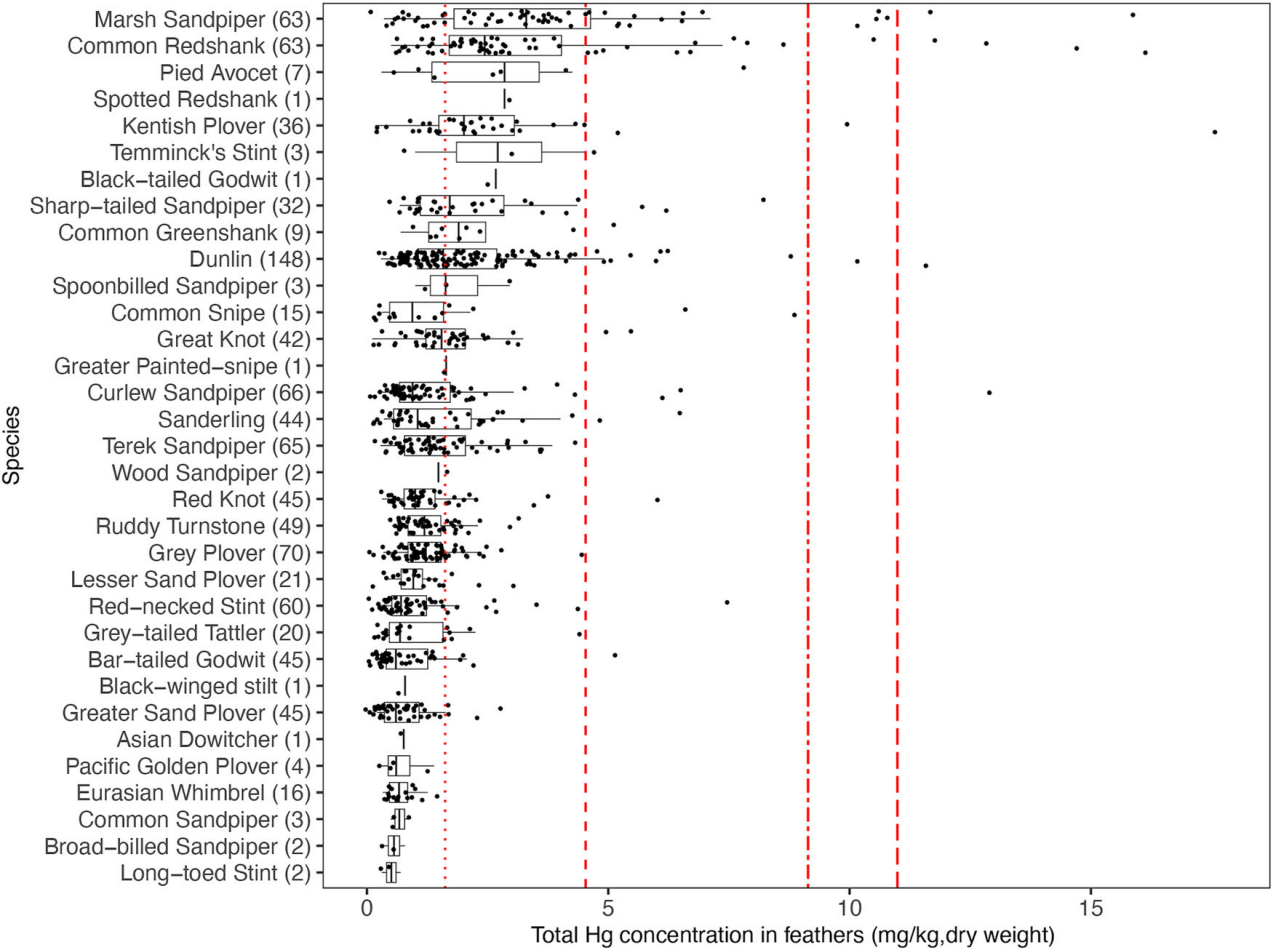
Total Hg (THg) concentration in feathers of different shorebird species along the East Asian-Australasian Flyway. Vertical red dashed lines show Hg toxicity thresholds^14,77^ from left to right, representing low risk (1.62 mg/kg), moderate risk (4.53 mg/kg), high risk (9.14 mg/kg), and severe risk (10.99 mg/kg). For each species, the number in parentheses indicates the sample size. Boxplots show median and 25% and 75% quartiles. Species are ranked by median feather THg concentrations. Scientific names can be found in Supplementary Data 1 and 2 (all species) and in Table 1. The x-axis is truncated at 18 mg/kg for clarity; one individual data point of 38.63 mg/kg from a Marsh Sandpiper is not shown.

Variations in feather THg concentrations among shorebird species were considerable (for summary statistics see Supplementary Table 1, Fig. 1). For example, the feather THg concentration for the Marsh Sandpiper *Tringa stagnatilis* (4.39 ± 5.35 mg/kg; median: 3.40 mg/kg, range: 0.35–38.63 mg/kg, *n* = 63) was approximately 5.7 times greater than that for the Bar-tailed Godwit (0.87 ± 0.77 mg/kg; median: 0.60 mg/kg, range: 0.30–4.85 mg/kg, *n* = 45) and Greater Sand Plover *Charadrius leschenaultii* (0.77 ± 0.55 mg/kg; median: 0.60 mg/kg, range: 0.21–2.5 mg/kg, *n* = 45). In addition, species belonging to the *Tringa* genus with a preference for inland wetlands and agricultural habitats, including the Marsh Sandpiper, Common Redshank *Tringa totanus*, Spotted Redshank *Tringa erythropus*, and Common Greenshank *Tringa nebularia*, had higher feather THg concentrations than most of the other genera. Also, our findings showed very little evidence of Hg threat in species such as the Eurasian Whimbrel *Numenius phaeopus*, Greater Sand Plover, Bar-tailed Godwit, Grey-tailed Tattler *Tringa brevipes*, and Red-necked Stint *Calidris ruficollis*.

Based on toxicity benchmarks, we found 62.80% of individual shorebirds (618 of 984) showed feather THg concentrations that were below the Hg toxicity threshold considered for adverse impacts (Table 1, Fig. 1), while 31%, 4.47%, 0.71% and 1.01% were within the low risk, moderate risk, high risk and severe risk categories, respectively. Among 16 species with adequate sample size (*n* ≥ 15), 5 species had less than or equal to ~50% of the sampled individuals within a no-apparent risk category: the Marsh Sandpiper (22.22%), Common Redshank (22.22%), Kentish Plover (33.33%), Sharp-tailed Sandpiper (50.00%) and Dunlin (50.68%). Notably, the Common Redshank and Marsh Sandpiper were standing out here, with ~50% of individuals at low, ~15% at moderate, ~1%–5% at high, and even ~5% at severe risk (feather THg concentrations $\ge \,$10.99 mg/kg). Of the seven individuals at severe risk, two were from the temperate zone, while the other five were from South China.

**Table 1:** Mercury-contamination associated risk levels for migratory shorebirds along the East Asian-Australasian Flyway

Species	Scientific name	Sample size	Risk category (%)				
			N	L	M	H	S
**Marsh Sandpiper**	***Tringa stagnatilis***	**63**	**22.22**	**50.79**	**15.87**	**6.35**	**4.76**
**Common Redshank**	***Tringa totanus***	**63**	**22.22**	**55.56**	**14.29**	**1.59**	**6.35**
**Kentish Plover**	***Charadrius alexandrinus***	**36**	**33.33**	**58.33**	**2.78**	**2.78**	**2.78**
**Sharp-tailed Sandpiper**	***Calidris acuminata***	**32**	**50.00**	**40.63**	**9.38**	**0.00**	**0.00**
**Dunlin**	***Calidris alpina***	**148**	**50.68**	**42.57**	**5.41**	**0.68**	**0.68**
Great Knot	*Calidris tenuirostris*	42	52.38	42.86	4.76	0.00	0.00
Sanderling	*Calidris alba*	44	61.36	34.09	4.55	0.00	0.00
Terek Sandpiper	*Xenus cinereus*	65	67.69	32.31	0.00	0.00	0.00
Curlew Sandpiper	*Calidris ferruginea*	66	72.27	22.73	3.03	0.00	1.52
Grey-tailed Tattler	*Tringa brevipes*	20	75.00	25.00	0.00	0.00	0.00
Ruddy Turnstone	*Arenaria interpres*	49	75.51	24.49	0.00	0.00	0.00
Grey Plover	*Pluvialis squalarola*	70	78.57	21.43	0.00	0.00	0.00
Red Knot	*Calidris canutus*	45	80.00	17.78	2.22	0.00	0.00
Red-necked Stint	*Calidris ruficollis*	60	86.67	11.67	1.67	0.00	0.00
Bar-tailed Godwit	*Limosa lapponica*	45	91.11	6.67	2.22	0.00	0.00
Greater Sand Plover	*Charadrius leschenaultii*	45	93.33	6.67	0.00	0.00	0.00
**All Species**		**984**	**62.80**	**31.00**	**4.47**	**0.71**	**1.01**

Species with adequate samples (n $\ge$ 15) are assigned to five risk categories (expressed in %) based upon feather-specific Hg effect thresholds^14,77^. The species are ordered from high to low based on the no apparent effect risk category. The top five species at highest risk are highlighted in bold. The overall risk for all species combined is presented in the last row.

*N* no apparent effect, *L* low risk, *M* moderate risk, *H* high risk, *S* severe risk.

Below, we provide the main findings of Hg exposure across the various regions for the five species from Table 1 with the highest Hg risk. For the remaining 11 species from Table 1, this detail is provided in the Supplementary Results of Supplementary Information Section. We present the species below in the order from high to low based on the no apparent effect risk (N) category.

#### Marsh Sandpiper

This species is currently listed as Least Concern according to IUCN Red List ^5^. It accumulates elevated feather THg in both its temperate breeding grounds in Russian Siberia (2.68 ± 2.21 mg/kg, median: 1.95 mg/kg, range: 0.35–7.21 mg/kg, *n* = 21 juveniles) and its wintering grounds in South China and northwest Australia (5.25 ± 6.21 mg/kg, median: 3.52 mg/kg, range: 0.60–38.63 mg/kg, *n* = 42 adults), yet feather THg was nearly two times higher in the wintering ground than breeding grounds (*F*_1,61_ = 10.19, *p* = 0.0022). Overall, 49 of the 63 samples (77.78%) were in at-risk toxicity categories, with 32 (50.79%), 10 (15.87%), 4 (6.35%), and 3 (4.76%) falling in the low, moderate, high, and severe risk categories, respectively. Notably, the seven adults at high and severe risk were all from Mai Po in South China.

#### Common Redshank

This species has an extremely large range and its conservation status is currently considered of Least Concern, exhibiting an unclear population trend^5,51^. The sample collection consisted of 33 juveniles thought to originate from the temperate zone (likely Mongolia and Northeast China), while 30 adults were thought to have grown their feathers in local capture sites of South China (28 in Mai Po and 2 in Leizhou). This species accumulated a concerningly high THg concentration at both the breeding grounds (3.48 ± 2.91 mg/kg, median: 2.32 mg/kg, range: 0.50–12.59 mg/kg, *n* = 33 juveniles) and non-breeding grounds (3.85 ± 3.80 mg/kg, median: 2.48 mg/kg, range: 0.72–15.94 mg/kg, *n* = 30 adults, *F*_1,61_ = 0.02, *p* = 0.89). Overall, 49 of the 63 (77.78%) Common Redshank samples were considered to be in risk categories with 35 (55.56%), 9 (14.29%), 1 (1.59%) and 4 (6.35%) within the low, moderate, high, and severe risk categories, respectively. Of the five individuals in the high and severe risk categories, two were from the Temperate zone, while three were from South China (2 in Mai Po and 1 in Leizhou).

#### Kentish Plover

This species currently has an unknown population trend^5^. Given its unclear moult ecology, only 18 of the 36 samples could be assigned with certainty from the low temperate to the subtropical zone of Asia. This species accumulated relatively high amounts of Hg at the breeding grounds (3.31 ± 4.29 mg/kg; median: 1.67 mg/kg, range: 0.24–17.83 mg/kg, *n* = 18). 24 (66.67%) of the 36 sampled individuals were within risk categories, with 21 (58.33%), 1 (2.78%), 1 (2.78%) and 1 (2.78%) within the low, moderate, high and severe-risk categories, respectively.

#### Sharp-tailed Sandpiper

This species is considered Vulnerable^5^. The juveniles from Lake Eda and Western Port likely originated from Arctic Russian Siberia, while adults from 80 Mile Beach and Port Phillip Bay reflect local contamination in northwest Australia and Victoria, Australia. This species accumulated significantly higher feather THg concentrations (*F*_1, 29_ = 11.71, *p* = 0.0018) in adults at their non-breeding grounds (2.86 ± 1.90 mg/kg; median: 2.25 mg/kg, range from 1.02 to 8.33 mg/kg, *n* = 21) compared to juveniles at the breeding grounds (1.31 ± 0.80 mg/kg; median: 1.11 mg/kg, range: 0.68–3.58 mg/kg, *n* = 11). In the Sharp-tailed Sandpiper, 16 (50.00%) of the 32 sampled individuals were within risk categories, with 13 (40.63%) and 3 (9.38%) of the studied population in the low risk and moderate risk categories, respectively, with no individuals falling in the high risk or severe risk categories.

#### Dunlin

Due to its relatively large population size and worldwide distribution, the Dunlin is considered a species of Least Concern despite evidence that the global population is declining^5^. Along the EAAF, the population trend is unknown. All samples were collected along the coast of China. Hg concentration in juveniles represented contamination from the mid temperate to Arctic region (Russia Siberia-Alaska, 1.62 ± 1.49 mg/kg; median: 1.24 mg/kg, range from 0.29 to 10.47 mg/kg, *n* = 81), while adults represented Hg contamination in temperate-Arctic (i.e., Yellow Sea to Arctic, 2.72 ± 1.85 mg/kg; median: 2.26 mg/kg, range from 0.55 to 11.36 mg/kg, *n* = 62), and in the Yellow Sea (2.25 ± 0.89 mg/kg; median: 2.09 mg/kg, 1.38 to 3.27 mg/kg, *n* = 5). 73 (49.32%) of the 148 Dunlin samples were within at-risk toxicity categories, with 63 (42.57%), 8 (5.41%), 1 (0.68%), and 1 (0.68%) in the low, moderate, high and severe risk categories, respectively.

### Factors contributing to Hg contamination in shorebirds

Overall, results from 16 candidate linear mixed-effects models (Table 2 and Supplementary Table 2) showed that variation in feather THg was importantly explained by region (weight: 1.00), with region included in all of the top 4 models (ΔAICc $\le$ 2). Habitat preference (weight: 0.32, included in 1 model), percentage of invertebrate diet (weight: 0.17, included in 1 model) and foraging stratum (weight: 0.15, included in 1 model) were additional factors but considerably less pronounced in explaining Hg contamination levels. These results from the model selection (region as the most influencing factor) were consistent with the previous results presented on a species basis, where significant differences in feather THg concentrations were found within species in various regions along the EAAF. Notably, one species group, i.e., the *Tringa* genus (e.g., the Marsh Sandpiper and the Common Redshank), faced a Hg threat, particularly at their wintering grounds in South China.

**Table 2:** Linear mixed-effects model selection for predicting feather total mercury (logHg: log_10_ transformed THg) for migratory shorebirds along the EAAF

Model	Intercept	df	logLik	AICc	ΔAICc	Weight
**logHg ~ Region** + **(1|Species)** + **(1|Year)**	$-$**0.02**	**8**	$-$**161.19**	**338.58**	**0.00**	**0.26**
**logHg ~ Region + Habitat Preference** + **(1|Species)** + **(1|Year)**	$-$**0.08**	**9**	$-$**160.28**	**338.80**	**0.22**	**0.23**
**logHg ~ Region + Diet** + **(1|Species)** + **(1|Year)**	$-$**0.06**	**9**	$-$**160.90**	**340.04**	**1.46**	**0.13**
**logHg ~ Region + Foraging Stratum** + **(1|Species)+ (1|Year)**	**0.00**	**9**	$-$**161.06**	**340.36**	**1.78**	**0.11**
logHg ~ Region + Foraging Stratum + Habitat Preference + (1|Species) + (1|Year)	$-$0.06	10	$-$160.17	340.65	2.07	0.09
logHg ~ Region + Diet + Habitat Preference + (1|Species) + (1|Year)	$-$0.09	10	$-$160.20	340.70	2.12	0.09
logHg ~ Region + Diet + Foraging Stratum + (1|Species) + (1|Year)	$-$0.04	10	$-$160.73	341.77	3.19	0.05
logHg ~ Region + Foraging Stratum + Habitat Preference + Diet + (1|Species) + (1|Year)	$-$0.07	11	$-$160.08	342.52	3.94	0.04
logHg ~ Habitat Preference + (1|Species) + (1|Year)	0.01	5	$-$185.23	380.54	41.96	0.00
**logHg** ~ **1** + **(1| Species)** + **(1|Year)**	**0.09**	**4**	$-$**186.42**	**380.89**	**42.31**	**0.00**
logHg ~ Diet + (1|Species) + (1|Year)	0.03	5	$-$185.93	381.95	43.37	0.00
logHg ~ Foraging Stratum + Habitat Preference + (1|Species) + (1|Year)	0.05	6	$-$184.92	381.95	43.37	0.00
logHg ~ Foraging Stratum + (1|Species) + (1|Year)	0.13	5	$-$186.05	382.18	43.60	0.00
logHg ~ Diet + Habitat Preference + (1|Species) + (1|Year)	$-$0.01	6	$-$185.07	382.25	43.67	0.00
logHg ~ Diet + Foraging Stratum + (1|Species) + (1|Year)	0.07	6	$-$185.50	383.12	44.54	0.00
logHg ~ Foraging Stratum + Habitat Preference + Diet + (1|Species) + (1|Year)	0.03	7	$-$184.72	383.58	45.00	0.00

Species and sampling years were included as a random effect. The number of observations used in all models was 742. Individuals without clear region information were omitted. The selected models (ΔAICc $\le$ 2) and the intercept-only model are depicted in bold.

**Fig. 2:**
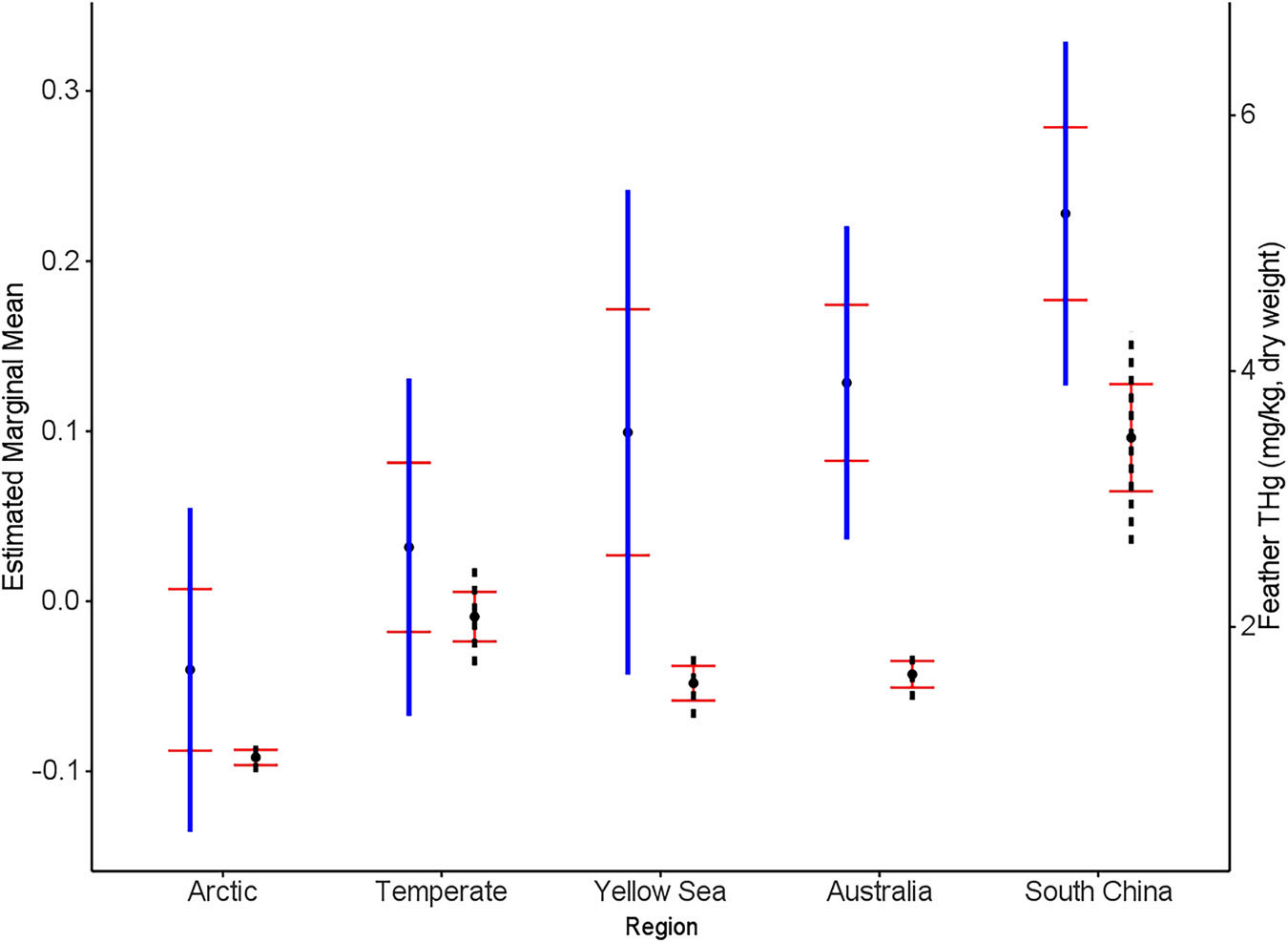
Hg concentration in feathers based on model estimations vs. observed calculations. The number of observations used here was 742. Left: the estimated marginal means (log_10_ transformed THg concentration) for the region on the full model (logHg ~ Region + Habitat Preference + Foraging Stratum + Diet + (1|Species) + (1|Year)). Right: the observed means of total Hg concentration in feathers. The standard errors of both means are indicated by red horizontal bars. The blue vertical lines indicate the estimated 95% CI while the black vertical dash lines indicate the observed 95% CI.

We used unconditional model averaging across all candidate models to evaluate all combinations of predictor variables (Table 3), showing that the region was significant (*p* $\le$ 0.05), while habitat preference, invertebrate percentage and foraging stratum were not significant at any level. We found that feather THg concentrations (log_10_-transformed estimated means depicted in Fig. 2 and Supplementary Table 3) were significantly higher in regions of South China (0.21, 95% CI: 0.09 to 0.33), Australia (0.13, 95% CI: 0.03 to 0.23) and the Yellow Sea (0.10, 95% CI: $-$0.05 to 0.25) compared to the temperate (0.02, 95% CI: $-$0.09 to 0.13) and Arctic zone ($-$0.03, 95% CI: $-$0.13 to 0.08). Specifically, no significant difference was found between breeding grounds (temperate vs. Arctic, *p* = 0.86). South China exhibited significantly higher estimated mean THg concentrations compared to the Arctic (*p* < 0.0001) and the temperate (*p* < 0.0001). Compared with the estimated marginal means for the region, our observed feather Hg data showed a similar trend (Fig. 2), except for Hg in temperate was the second highest group. This could be driven by four individuals (two Common Redshank and two Kentish Plover) with extremely high Hg concentrations (11.08–17.83 mg/kg) originating from the temperate region. Together, our results suggest that region was the most important predictor of Hg in feathers.

**Table 3:** Model averaged coefficients in the candidate model set, predicting feather mercury (log_10_ transformed THg concentrations) for migratory shorebirds along the EAAF

Explanatory variable	Estimate	Std. Error	Adjusted SE	z value	Pr (>|z|)
(Intercept)	$-$0.05	0.07	0.07	0.73	0.47
Region: Temperate	0.06	0.05	0.05	1.16	0.25
Region: Yellow Sea	0.13	0.07	0.07	1.84	0.07
Region: Australia	0.16	0.03	0.03	4.94	0.00
Region: South China	0.24	0.05	0.05	5.03	0.00
Habitat Preference: NCO	0.04	0.07	0.07	0.64	0.53
Diet: B	0.01	0.05	0.05	0.30	0.76
Foraging Stratum: Surface	$-$0.01	0.04	0.04	0.25	0.80

The number of observations used for all models was 742. Habitat Preference: each species’ typical dependency on coastal habitats during the non-breeding season (NCO: non-coastal obligate, <100% use of coastal habitats; CO: coastal obligate, 100% use). Foraging Stratum: the prevalence of foraging time on or just below the water surface, indicated by the estimated use percentage (surface: <50%; depth: 50%–100%). Diet: the percentage of consumed invertebrates (A: 50%–79%; B: 80%–100%).

For the other three explanatory variables investigated, there were no significant differences between factor levels (Supplementary Figs. 1–3). Specifically, the estimated marginal means (emmeans) of log_10_-feather THg concentrations in foraging stratum showed no difference (deep: 0.11, 95% CI: $-$0.025 to 0.24; surface: 0.070, 95% CI: $-$0.050 to 0.19; *p* = 0.66; Supplementary Fig. 1). Within habitat preference, the emmean of log_10_-feather THg concentrations in non-coastal obligates (NCO: 0.13, 95% CI: 0.011 to 0.25) was approximately three times higher than that in coastal obligates (CO: 0.043, 95% CI: $-$0.092 to 0.18), yet remained non-significant (*p* = 0.29, Supplementary Fig. 2). For diet, no difference was found in the emmean of log_10_-feather THg concentrations of the consumption of 50%–79% invertebrates (A: 0.071, 95% CI: $-$0.070 to 0.21) and consumption of 80%–100% invertebrates (B: 0.10, 95% CI: $-$0.011 to 0.22; *p* = 0.69; Supplementary Fig. 3).

## Discussion

To the best of our knowledge, this is the first study that combines knowledge of moult strategy and life history with a standardised sampling protocol to assess Hg contamination in migratory shorebirds across an entire flyway at an intercontinental scale. Nearly 1000 individuals from 33 shorebird species were sampled, and their feathers were analysed for Hg concentration. These samples reflected Hg contamination across multiple regions of the world within the breeding and non-breeding stages of the globally important EAAF. Based on THg in feathers, our findings indicated regional differences are more important than other factors (diet, habitat preference and foraging stratum). Specifically, non-breeding grounds in South China, the Yellow Sea and Australia had a more pronounced Hg contamination level, twice as high as on the breeding grounds in the Arctic and the temperate zone.

Over one-third of shorebird individuals across all species were categorised above toxicity benchmarks for adverse impacts caused by Hg. Specifically, 31% of individuals fell within low risk, 4.47% within moderate risk, and 0.71% and 1.01% within high risk and severe risk categories, respectively. Feather THg concentrations underscored varying risks among shorebird species along the EAAF. On a broad scale, species exhibited fluctuating percentages within the low-risk (6%–60%) and moderate-risk (0%–15%) categories, with a narrower range of 0%–6% in the high and severe-risk categories. Notably, five species displayed individuals surpassing the high and severe-risk benchmarks, with ~58% representing heightened risks in South China. In comparison, employing identical feather-specific THg effect thresholds, Chastel et al.^14^ observed a distinctive distribution among individuals in the Arctic. A significant proportion fell within the low-risk category (70%–100%), with 3%–30% classified as moderate risk. Conversely, a minimal proportion (<3%) fell into the high risk category (predominantly originating from Barrow, Alaska), and none were categorised as severe risk. Overall, compared with the Arctic populations, our study populations along the EAAF showed no difference in the percentages of low and moderate risk categories, while disproportionately higher rates in high and severe risk categories, particularly for individuals moulting in South China.

According to our findings, the region where birds had been growing their feathers was the strongest factor contributing to explaining variation in Hg loads, whereas the influence of species characteristics (habitat preference, diet, and foraging stratum) was much less pronounced. The highest percentage of individuals categorised as high and severe risk was observed in Mai Po, followed by Leizhou, both of which are located in South China. This suggests that these areas in South China have a larger proportion of individuals facing considerable risks than other locations. As one of 21 Ramsar sites (Wetlands of International Importance) in China, Mai Po serves as a key site for migratory shorebirds as well as other waterbirds, including at least 22 globally threatened species^52^. Situated close to two enormous cities (Shenzhen and Hong Kong) on the coast of the Pearl River Estuary, the levels of Hg in sediments of coastal areas of Hong Kong have decreased after the early 1990s due to strengthened pollution control^53^. Yet, recent studies have shown that mangrove ecosystems and adjacent mudflats in Shenzhen still exhibit significantly elevated Hg sediment levels^54^. Mangroves are believed to serve as a sink that retains and reduces the remobilisation of metals in sediment^54^. Our study indicates the intricate mechanisms involved in the bioavailability and bioaccumulation of Hg in shorebird food webs, emphasising the critical need for long-term biomonitoring to accurately assess the ecological risk to migratory shorebirds and long-term clean-up efforts to reduce the pollutant exposure in polluted areas.

Our study found that certain species within the *Tringa* genus, such as the Common Redshank and Marsh Sandpiper, exhibited high levels of Hg in feathers grown in both juveniles at breeding grounds and adults at non-breeding grounds, suggesting that they face a heightened risk of Hg contamination throughout their annual cycle. These species typically feed along channels, vegetated margins of salt marshes or mangroves^55^ and are frequently exposed to bioavailable methylmercury^56^. Taken together, our findings suggest that non-coastal obligate species frequently encountered in freshwater habitats throughout their annual cycle^1^, such as the Marsh Sandpiper, Common Redshank, Spotted Redshank, Pied Avocet *Recurvirostra avosetta*, Black-tailed Godwit, Temminck’s Stint *Calidris lemminckii* and Sharp-tailed Sandpiper may be at the highest risk of Hg exposure. Additionally, one of the three sampled Spoon-billed Sandpipers *Calidris pygmaea* exhibited elevated feather THg concentrations close to the reproduction threshold of 3 mg/kg^57^ in the Yellow Sea. This finding emphasises the need for better management and clean-up of industrial contaminants in this region, not only of Hg but also of persistent organic pollutants (POPs) such as flame retardants and other industrial chemicals (e.g., PCBs, PFAS) and agricultural chemicals (e.g., neonicotinoids) that are also present at high levels in this particular region^58–61^.

Given the complicated processes involved in the uptake, bioaccumulation, and biomagnification of Hg in foodwebs across various ecosystems^62^, current research efforts are focusing on understanding why Hg emission reduction under the Minamata Convention on Mercury have failed to improve trends of Hg exposure among monitored biota, such as fish and other aquatic wildlife^63^. Biomonitoring in understudied but quickly declining shorebird populations that inhabit wetland habitats regularly contaminated by Hg should represent an essential part of evaluating the effectiveness of the Convention in meeting its goals (http://www.mercuryconvention.org). While previous research has examined shorebirds from a wide region across the Arctic (but see review in ref. ^14^), our study can aid in identifying biological hotspots (South China) and sentinel species (the *Tringa* genus) for Hg biomonitoring programmes^37^. Further research is needed on the source, bioaccumulation, and biomagnification of Hg along shorebird food webs, as well as effects on behavioural ecology and survival. Moreover, shorebirds within their migratory flyways encounter a spectrum of pollutants beyond Hg, constituting a complex amalgamation of toxic substances prevalent in the contaminated mudflats and river ecosystems where these avian species forage^10,39,64^. This repertoire of contaminants encompasses a range of metals, elements and organic pollutants, which, similar to Hg, become integral components of the shorebirds’ dietary intake. This is attributed to the bioaccumulation of these substances in invertebrate organisms and their recalcitrance to degradation.

Our study employed a novel approach to evaluate Hg contamination in migratory shorebirds along the EAAF using standardised feather sampling. While our methodology has proven effective, it has some limitations and uncertainties. One such limitation is our reliance on feather moult ecology as the basis for our research design. More specifically, we cannot entirely rule out that some of the measured Hg contamination originates from stop-over sites that birds visited during their southward migration, albeit moult is typically limited during migration (but see in ref. ^65^). Secondly, the scarcity of detailed information regarding Hg patterns in moult ecology in shorebirds limits the applicability of our sampling protocol. The impact of age on Hg accumulation in feathers is still not fully understood. In the case of the Common Redshank, a species with ～8% of the population at high and severe risks of Hg contamination, one individual (Flag DN, ring number DK61361) in Mai Po was identified as being at least 11 years old. Conducting further research on species that undergo moulting in the same area and monitoring individuals that have been recaptured would provide a better understanding of how age affects Hg accumulation in feathers. Nevertheless, feathers provide a practical means to conduct large-scale spatiotemporal investigations of Hg contamination for migrant species travelling across continents during breeding, stopover, and wintering stages^66^. This is particularly true for species for which common biological metrics such as blood are difficult to sample in large enough amounts for analysis due to their small body size and threatened status, as is the case with many shorebird species. To overcome these research challenges, a standardised protocol should be followed with routine ringing exercises.

Our study provides the first, unique and detailed insight into Hg contamination across various declining shorebird species along the EAAF during their life cycle. Migratory shorebirds in the non-breeding range, especially in South China, showed higher Hg loads than those from other regions. Populations of the *Tringa* genus, particularly in South China, had the highest risk from Hg contamination. This study delineates prominent contamination hotspots within the EAAF attributable to elevated Hg emissions. Urgent and decisive measures are imperative to curtail these emissions and initiate recovery in regions posing the highest risk. Failure to address this pressing issue promptly may result in further declines in shorebirds and other species. Overall, continued international collaborations with local researchers banding birds along the EAAF where standardised feather sampling protocols are in place may be of assistance to enhance our ability to act towards the conservation of this increasingly imperilled suite of shorebird migrants along the EAAF.

## Methods

### Sampling locations and study species

**Fig. 3:**
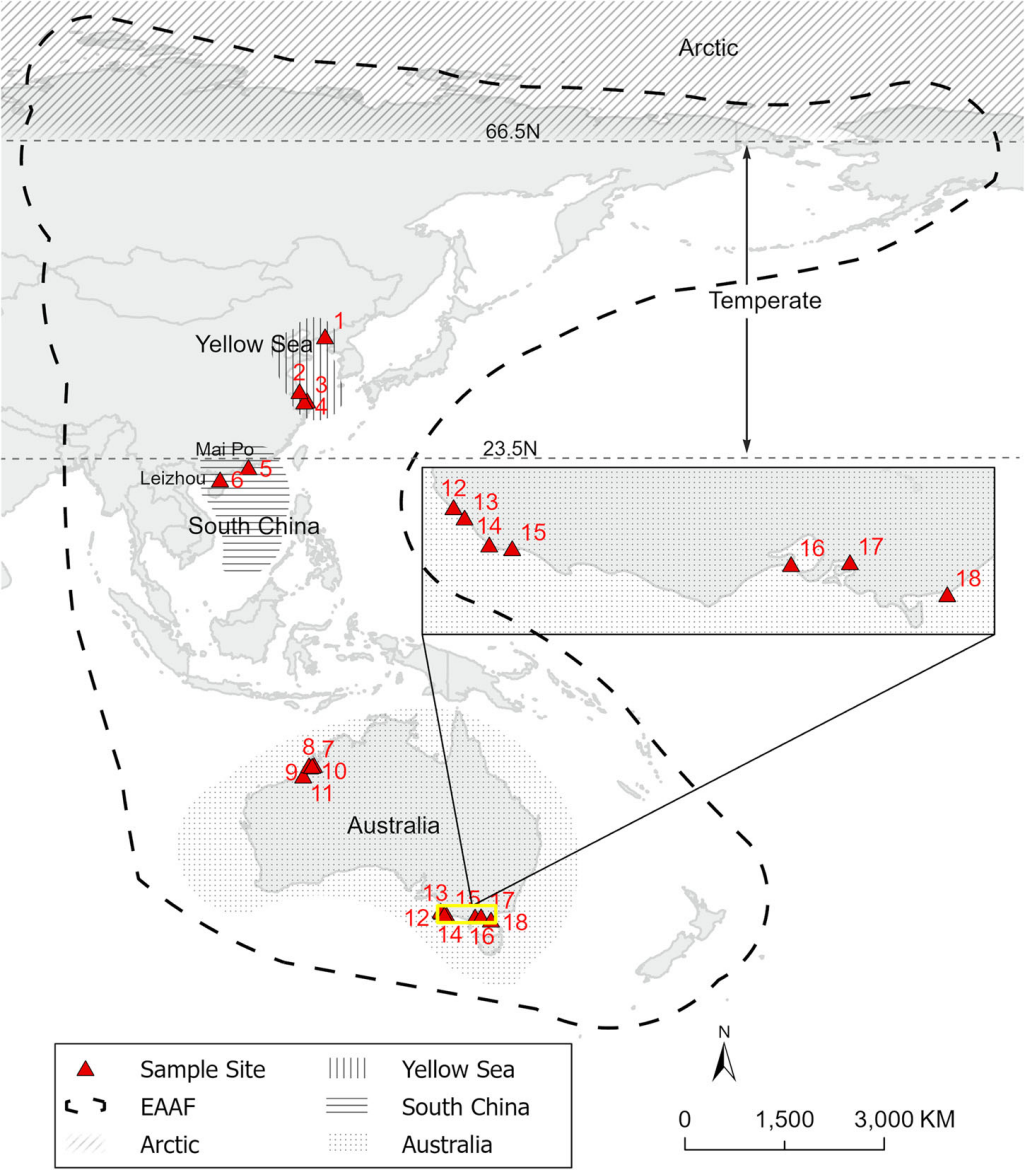
Sampling sites along the East Asian-Australasian Flyway. 1: Yalujiang; 2: Tiaozini; 3: Chongming Dongtan; 4: Yangkou; 5: Mai Po; 6: Leizhou; 7: Taylors Lagoon; 8: Coconut Well Beach; 9: Lake Eda; 10: Roebuck Bay; 11: 80 Mile Beach; 12: Beachport; 13: Canunda; 14: Nene Valley; 15: Brown Bay; 16: Port Phillip Bay; 17: Western Port; 18: Corner Inlet. Sites are numbered in order of latitude. Map layers were provided by Esri, FAO, NOAA, and USGS (ArcGIS Pro 3.0.2).

Samples were collected from 18 sites in China and Australia (Fig. 3). Species sampled (*n* = 33) were selected based on their distribution along the coasts in China and Australia, moult ecology, and ability to catch them. Along the Chinese coast, migratory shorebirds (*n* = 622) were captured using mist nets or clap nets and sampled during non-breeding periods (from January to April and August to December) between 2019 and 2022. Six bird ringing stations were included: Yalujiang Coastal Wetland (Yalujiang) in Liaoning at the northern part of the Yellow Sea; Tiaozini and Yangkou in southern Jiangsu at the southern part of the Yellow Sea; Chongming Dongtan National Nature Reserve in Shanghai (Chongming Dongtan); Mai Po in Hong Kong and Leizhou Peninsula in Guangdong of South China. In Australia, birds (*n* = 362) were captured by cannon nets or mist nets between 2004 and 2016. Twelve sampling sites were included in Australia. There were five sites in northwest Australia: 80 Mile Beach and 4 sites near Broome (Coconut Well Beach; Lake Eda; Roebuck Bay and Taylors Lagoon). There were seven sites in southeast Australia: Corner Inlet and two sites (Port Phillip Bay and Western Port) near Melbourne, and four sites (Beachport, Brown Bay, Canunda and Nene Valley) in Limestone Coast. Aside from feathers sampled from live birds, feathers were also collected from accidental catch casualties and carcasses encountered opportunistically in the field. For further details of study species, sample sizes, capture locations, and collected periods, see Supplementary Data 1 and Supplementary Data 2.

For each species, we created a variable “habitat preference” reflecting each species’ typical dependency on coastal habitats during the non-breeding season: i.e., non-coastal obligate (NCO, <100% use of coastal habitats) and coastal obligate (CO, 100% use, in ref. ^67^. For each species, foraging stratum and diet were obtained from expert descriptions in published literature and translated into a coarse proportional assignment^68^. Specifically, the foraging stratum was determined based on the prevalence of foraging time on or just below the water surface (<5 in.), indicated by the estimated use percentage (surface: <50%; depth: 50%–100%), while the diet was characterised by estimated percentage of consumed invertebrates (A: 50%–79%; B: 80%–100%).

### Feather sampling

Previous studies indicate during moult, growing feathers reflects dietary Hg well^47^ and a large proportion (up to 90%) of Hg burden is excreted via it^69,70^. Given that shorebirds occupy a relatively low trophic position, we assumed that age did not affect Hg bioaccumulation, and thus any observed differences in Hg concentrations in feathers between age groups would merely reflect variation in the site of feather growth associated with variation in local contamination levels. Accordingly, along this flyway, juveniles generally replace flight feathers at breeding/natal grounds, while adults mostly replace flight feathers at non-breeding grounds (including wintering and stop-over sites), except Dunlins that initiate primary moult at the breeding ground^71–74^. Thus, feather Hg in juveniles and adults indicated Hg contamination at breeding grounds and non-breeding grounds, respectively, unless stated otherwise. Feathers from individuals caught while in active moult generally replace flight feathers at the catch-site, and their feather Hg accordingly represents the catch-site contamination. In general, if available and fully grown, we collected the 6th primary covert (PC6, Supplementary Fig. 4), which moults at the same time as the 6th primary feather^75^. Because all of our studied species have sequential primary moult, the growth of PC6 will occur around or shortly after the feather half of the depuration curve was reached. Indeed, in songbirds, the feather Hg concentrations half peak value of depuration was reached in between the moult of primary 4th and primary 5th (in ref. ^47^, mentioned in SI 2.2). Thus, PC6 Hg concentrations could represent both the near-half depuration value and the median THg value of the whole primary coverts (article in writing) since shorebirds have ten primaries instead of nine in songbirds. For individuals in active primary moult with old PC6, the nearest most recent completely-grown primary covert was collected. Feathers were stored in paper envelopes or plastic zip-loc bags at room temperature. Birds were assigned to age classes (juvenile or adult), which was determined mainly by capture date, location and plumage status, using criteria outlined in refs. ^71,73^. For example, adult birds captured in the Yellow Sea during autumn without active moult status would have uncertain moulting origin while those captured in South China or Australia would be likely to initiate moult at those sites. These moulting regions mainly encompassed the breeding grounds (including the Arctic and temperate regions), and the non-breeding grounds (encompassing the Yellow Sea, South China, and Australia). Details can be found in Supplementary Data 2 and are summarised in Supplementary Table 4.

### Mercury measurement

Prior to analysis, whole feathers were rinsed with 1% acetone and deionised water, then dried at ambient temperature overnight. The feather THg concentration was determined using a Direct Mercury Analyser (DMA 80, Milestone Inc., Italy, detection limit: 0.005 ng), following the US Environmental Protection Agency Method 7473^76^. Samples (weight range between 0.0010 to 0.0900 g) were measured at the State Key Laboratory of Environmental Geochemistry in Guiyang, China. Samples were weighed on a clean nickel sample boat at a balance of 0.0001-gram precision. Laboratory quality control samples included a blank (no nickel boats), a method blank (empty nickel boats with aluminium foil), and certified reference materials (IAEA-86, Human Hair, International Atomic Energy Agency, Vienna, Austria; DORM-4, fish protein, National Research Council Canada), and a duplicate feather sample with each batch containing 15 or fewer samples. The detection limit for Hg in feathers using DMA 80 was 0.0008 mg/kg. Relative percentage difference (mean ± sd) for duplicate samples was 12.13 ± 11.25% (*n* = 66). Recovery of IAEA-86 was 93.40 ± 10.34% (*n* = 40), while for DORM-4 it was 96.46 ± 5.83% (*n* = 56).

### Risk assessment

Our assessment of population-level impacts of Hg contamination used the same risk categories used in studying the Hg contamination in Arctic shorebirds^14^. Specifically, the five thresholds were based on toxicity benchmarks established for bird blood^77^ and then converted into equivalent body feather Hg concentrations by ref. ^14^. The details of the five risk thresholds are no apparent effect (<1.62 mg/kg); low risk (1.62–4.53 mg/kg; health, physiology, behaviour and reproduction affected), moderate risk (4.53–9.14 mg/kg; severe impairment to health and reproduction), high risk (9.14–10.99 mg/kg; physiological and reproductive effects, often complete reproductive failure), and severe risk ($\ge$10.99 mg/kg; severe physiological and reproductive effects, including adult mortality).

### Statistics and reproducibility

Firstly, for individuals (*n* = 876) with clear grounds for life stage, to explore the difference between locations (breeding vs. non-breeding) without the inclusion of habitat preference, diet and foraging stratum, an analysis was conducted where log_10_ transformed feather THg (logHg) was the dependent variable and location (breeding ground vs. non-breeding ground) was the fixed factor explanatory variable, with species and year of sample collection as random factors in a linear mixed-effects model (anova function for results). Furthermore, for each individual species with a sample size ≥15, we used One-Way ANOVA (aov function, Type I sums of squares and summary function) to determine whether there was a difference between breeding ground and non-breeding ground.

Secondly, to determine factors contributing to variation in feather THg concentrations along the EAAF, we conducted a series of linear mixed-effects models for individuals with a clear moult region (*n* = 742, Table 2). In this analysis, we thus omitted individuals with either unclear moult areas or broad moult origins, such as unidentified areas between Southeast Asia and Australia, and only examined individuals from the five regions specified previously (Fig. 3). To avoid pseudoreplication, all models included species and year as random effects. The potential factors contributing to variation in feather THg concentrations include: geographic region of feather growth (five categories), habitat preference (two categories), foraging stratum (two categories) and diet (two categories). The detailed sub-categories of fixed effects corresponding to each species are presented in Supplementary Data 2.

The interaction effects were not considered here since it was out of the scope of this study. We tested and detected no multicollinearity among these variables based on the VIF scores in the full model of logHg ~ Foraging Stratum + Region + Habitat Preference + Diet + (1|Species) + (1|Year), details can be found in Supplementary Table 5. Models were run if VIF < 6. We included all combinations of predictor variables in candidate models. We used the Akaike Information Criterion adjusted for small sample sizes (AICc) to rank models, wherein we considered models with ΔAICc $\le$ 2 only^78,79^. Support for each model was evaluated using the Akaike weight, which represents the relative likelihood of the model relative to all other models in the candidate set. Within the candidate model set, we applied variable importance weights to assess an individual variable’s relative importance and we did model averaging using all candidate models for model averaged coefficients.

Within the full linear mixed-effects model (logHg ~ Region + Habitat Preference + Foraging Stratum + Diet + (1|Species) + (1|Year)), the estimated marginal means (i.e., emmeans) were calculated and plotted. Specifically, we applied the modavg function to aggregate the selected variable across the entire candidate model set, disregarding ∆AICc and model weight considerations. Following this, we ran the full model to show the concentration of feather Hg at each combination of the factors, the post hoc pairwise comparison for the main factor(s), and then plotted estimated marginal means with error bars and 95% confidence intervals (CI).

THg concentrations were log_10_ transformed to meet the assumption of normality. All analyses were performed in R statistical software^80^, using packages ‘performance’ (check_collinearity function, version 0.10.8^81^), ‘ggplot2’^82^, ‘lme4’ (lmer function, version 1.1-29^83^), ‘MuMIn’ (model.sel function, version 1.46.0^84^) and ‘emmeans’ (model.avg function, version 1.8.2^85^). Values were shown as mean ± s.d. (median, range, sample size) and estimates from the linear mixed-effects models were shown as mean and 95% CI.

### Reporting summary

Further information on research design is available in the Nature Portfolio Reporting Summary linked to this article.

### Supplementary information

Supplementary Information

Description of Additional Supplementary Files

Supplementary Data 1

Supplementary Data 2

Reporting Summary

## Data Availability

All data generated or analysed and the source data behind the figures can be found in Supplementary Data 1.
