# Supplementary material for: Mercury contamination is an invisible threat to declining migratory shorebirds along the East Asian-Australasian Flyway
Source: Commun Biol. 2024 May 16;7:585. doi: 10.1038/s42003-024-06254-x (PMC11098816; doi:10.1038/s42003-024-06254-x)
Supplement: Supplementary file 1 — Supplementary Information [file 42003_2024_6254_MOESM1_ESM.pdf]

Supplementary Information Section

Mercury contamination is an invisible threat to declining migratory shorebirds along the East Asian-Australasian Flyway

Yanju Ma [1,2,3], Chi-Yeung Choi [1,4*], Lihai Shang [5], Marcel Klaassen [6], Zhijun Ma [7], Qing Chang [8], Veerle L. B. Jaspers [9], Qingquan Bai [10], Tao He [11], Katherine K-S. Leung[12], Chris J Hassell [13], Roz Jessop [14], Luke Gibson [1*]

[1] School of Environmental Science and Engineering, Southern University of Science and Technology, Shenzhen, China

[2] Key Laboratory of Ecology of Rare and Endangered Species and Environmental Protection (Guangxi Normal University), Ministry of Education, Guilin 541006, Guangxi, China

[3] Guangxi Key Laboratory of Rare and Endangered Animal Ecology, College of Life Sciences, Guangxi Normal University, Guilin 541006, Guangxi, China

[4] Environmental Research Center, Duke Kunshan University, Kunshan 215316, Jiangsu, China

[5] State Key Laboratory of Environmental Geochemistry, Institute of Geochemistry, Chinese Academy of Sciences, Guiyang 550081, Guizhou, China

[6] School of Life and Environmental Sciences, Deakin University, Geelong, VIC, Australia

[7] Ministry of Education Key Laboratory for Biodiversity Science and Ecological Engineering, Coastal Ecosystems Research Station of the Yangtze River Estuary, Institute of Biodiversity Science, School of Life Sciences, Fudan University, Shanghai, 200433, China

[8] Nanjing Normal University, Nanjing 210024, Jiangsu, China

[9] Department of Biology, Norwegian University of Science and Technology (NTNU), Trondheim 7491, Norway

[10] Dandong Forestry and Grassland Development Service Center, Dandong 118000, Liaoning, China

[11] Zhanjiang Mangrove National Nature Reserve Bureau, Zhangjiang 524000, Guangdong, China

[12] Hong Kong Waterbirds Ringing Group, Mai Po Nature Reserve, Mai Po, Hong Kong, China

[13] Australian Wader Studies Group, Curtin, ACT 2605, Australia

[14] Victorian Wader Study Group, Thornbury, VIC 3071, Australia

* Corresponding author. E-mail address: biodiversity@sustech.edu.cn; choimo@yahoo.com

Supplementary Results

Species variations in feather THg concentrations and ecological risks along the EAAF

Great Knot. This species is endemic to the EAAF and has become Endangered owing to recent evidence showing a rapid population decline due to more than half of intertidal stopover habitat loss in the Yellow Sea region of East Asia[1]. The juveniles sampled from Roebuck Bay and Yalujiang are most likely breeding in Russian Siberia in the Arctic. We recorded that the feather THg concentrations in juveniles representing Hg contamination in Russian Siberia (1.87 ± 1.40 mg/kg; median: 1.50 mg/kg, range: 0.11 to 5.33 mg/kg, n = 19) showed no significant difference with feather THg concentrations in adults at Australian non-breeding grounds (1.68 ± 0.65 mg/kg; median: 1.75 mg/kg, range: 0.35 to 2.93 mg/kg, n = 23 adults; $F_{1,40} = 0.18$, $p = 0.68$). Overall, 22 (52.38%), 18 (42.86%) and 2 (4.76%) of the 42 samples were at no apparent effect, low and moderate risk categories, no individuals fell in neither high nor severe risk categories. The data of THg in feathers from other regions were not available.

Sanderling. This species is classified as Least Concern[1]. The feather THg concentrations in adults at non-breeding grounds (north-west Australia and south-east Australia, 1.88 ± 1.39 mg/kg; median: 1.82 mg/kg; range: 0.35 to 6.27 mg/kg; n = 30), was significantly higher than the feather THg concentrations in juveniles at natal grounds of Russian Siberia (0.81 ± 0.40 mg/kg; median: 0.72 mg/kg, range: 0.43 to

1.75 mg/kg, n = 10; $F_{1,42}$ = 4.57, p = 0.038). In Sanderling, 27 (61.36%) of the 44 sampled individuals showed no apparent effect risk to Hg, while 15 (34.09%) and 2 (4.55%) were at low and moderate risk category, respectively.

Terek Sandpiper. This species is classified as Least Concern, but the population trend appears to be decreasing[1]. The juveniles were most likely born in the temperate zone of Russian Siberia, while the adults mainly reflected local contamination at non-breeding sites (Mai Po and Yangkou) and 80 Mile Beach, north-west Australia. Notably, except for 7 individuals without clear moulting region, this species experienced no difference in Hg accumulation ($F_{1,56}$ = 0.39, p = 0.54) in both juveniles from natal grounds (1.52 ± 1.10 mg/kg; median: 1.30 mg/kg, range: 0.28 to 4.08 mg/kg, n = 34) and adults at non-breeding grounds (1.50 ± 0.82 mg/kg; median: 1.28 mg/kg, range: 0.51 to 3.58 mg/kg, n = 24). 44 (67.69%) of the 65 sampled individuals were within the no suspected risk category, while 21 (32.31%) were within the low-risk category.

Curlew Sandpiper. This species is listed as Near Threatened owing to a global population decline, particularly along the EAAF, with a steep population decline of over 50%[1]. We sampled 66 individuals here who were thought to breed in Russian Siberia in the Arctic during winter in Australia (Victoria and north-west Australia), and 36 individuals caught at Mai Po growed their feathers at a wide range of south-east Asia or Australia. Notably, this species accumulated significantly higher Hg contamination

in adults at their non-breeding grounds (0.77 ± 0.32 mg/kg; median: 0.70 mg/kg; range: 0.32 to 13.13 mg/kg, n = 55) compared to juveniles from their natal/breeding grounds (1.70 ± 2.00 mg/kg; median: 1.09 mg/kg; range: 0.39 to 1.36 mg/kg, n = 11, $F_{1, 64}$ = 5.22, p = 0.026). All juveniles from the Arctic-high region belong to the low/no apparent effect risk group. At Australian non-breeding grounds, compared to 80 Mile Beach (1.41 ± 0.48 mg/kg; median: 1.36 mg/kg, range: 0.80 to 2.35 mg/kg, n = 10), 2 to 3 times higher feather THg concentrations were found in Corner Inlet (5.37 ± 5.56 mg/kg; median: 3.94 mg/g, range: 0.46 to 13.13 mg/kg; n = 4) and Port Phillip Bay (3.05 ± 2.29 mg/kg; median: 2.30 mg/kg, n = 5, 0.58 to 6.45 mg/kg), indicating elevated Hg risk may exist at Victoria, Australia. Overall, 48 (72.72%), 15 (22.73%), 2 (3.03%), 0 (0%) and 1 (1.52%) of the 66 individuals were within no apparent effect, low, moderate, high and severe risk categories, respectively.

Grey-tailed Tattler. This species is currently listed as Near Threatened owing to evidence that it is undergoing a moderately rapid population decline, driven by multiple human-induced factors such as habitat loss[1]. Both juveniles and adults were collected from Broome. Except for 1 individual without a clear feather-grown region, they are likely reflecting their natal grounds at Russian Siberia (0.95 ± 1.28 mg/kg; median: 0.41 mg/kg; range: 0.28 to 4.14 mg/kg, n = 9 juveniles) and overwintering grounds mainly in north-west Australia (Broome, 1.03 ± 0.62 mg/kg; median: 0.75 mg/kg; range: 0.50 to 2.25 mg/kg, n = 10 adults). No significant differences were found between feather THg concentrations in breeding and non-breeding grounds ($F_{1,18}$ = 2.21, p = 0.15).

Among 20 sampled individuals, 15 (75%) and 5 (25%) were within no apparent effect risk and low risk category, respectively. No individuals were in any other higher-risk categories. No other data exist for other regions.

Ruddy Turnstone. This species is listed as Least Concern according to the IUCN Red List criteria[1]. We collected juveniles in Yalujiang, Broome, and Southeast Australia. The feather THg concentrations of juveniles likely presented the local contamination of the high-altitude Arctic when adult feather THg concentrations reflected the local contaminations in Australia, including south-east Australia and north-west Australia. Notably, this species experienced no difference in Hg accumulation ($F_{1,47} = 2.82$, $p = 0.10$) in both adults at non-breeding grounds (Australia: 1.13 ± 0.42 mg/kg; median: 1.26 mg/kg, range: 0.52 to 1.86 mg/kg; n = 22) and juveniles at natal grounds (Russian Siberia- Alaska, 1.39 ± 0.64 mg/kg; median: 1.08 mg/kg, 0.66 to 3.16 mg/kg; n = 27). Overall, 37 (75.51%) of the 49 sampled individuals showed no apparent effect risk to Hg, while 12 (24.48%) were within the low-risk category. No current studies of this species are found in other locations worldwide.

Grey Plover. This species is currently listed as Least Concern[1]. The juveniles likely reflected their natal grounds in Russian Siberia in the Arctic. The average feather THg concentration in juveniles from Russian Siberia is 1.23 ± 0.82 mg/kg (median: 1.06 mg/kg; range: 0.34 to 4.36 mg/kg, n = 25), while similar feather THg concentration in adults was found at the non-breeding grounds in South China and Australia (1.30 ± 0.51

mg/kg; median: 1.27 mg/kg; range: 0.43 to 2.67 mg/kg, n = 45; $F_{1, 68} = 1.06$, $p = 0.31$). Overall, 55 (78.57%) of the 70 sampled individuals fell in the no apparent effect risk category, while 15 (21.43%) individuals were in low risk category. No individuals were in other higher risk categories. No other data exist for other regions.

Red Knot. This species is listed as Near Threatened[1]. We sampled adults from Broome, Port Phillip and the feather THg concentrations represented local signals. Juveniles captured at Broome, Port Phillip, Yalujiang and Leizhou likely originated from Russian Siberia in the Arctic. Notably, this species accumulated significantly higher feather THg concentrations ($F_{1, 43} = 19.58$, $p < 0.00001$) in adults at non-breeding grounds (1.79 ± 1.29 mg/kg; median: 1.43 mg/kg, range: 0.37 to 6.28 mg/kg, n = 22), compared to juveniles at their breeding grounds (0.83 ± 0.28 mg/kg; median: 0.79 mg/kg, range: 0.31 to 1.41 mg/kg, n = 23). Previous research focuses on this species conducted mainly in Delaware Bay, United States (mean feather THg concentrations: 0.47 to 1.16 mg/kg, but also Pertuis Charentais in France (mean feather THg concentration: 1.26 mg/kg, range: 0.42 to 4.45 mg/kg, n = 15; details see in ref. [2–6]). Thus, Red Knot along the EAAF faced similar risks for Hg-associated effects compared with other studies. A large proportion, 36 (80%) of the 45 sampled individuals, fell in the no apparent effect risk category, while 8 (17.78%) and 1 (2.22%) were at low risk and moderate risk categories, respectively.

Red-necked Stint. The species are listed in the Near Threatened category[1]. We collected juveniles from the Southern Yellow Sea (Chongming Dongtan, Tiaozini, Yankou) and the South China coast (Leizhou) and the adults from Australian wintering sites (Roebuck Bay, Corner Inlet and Port Phillip Bay). The juveniles were likely to breed in Russian Siberia and the Arctic, while the adults reflected local contamination in South China and Australia. Notably, this species experienced no difference ($F_{1,58}$ = 2.36, p = 0.13) in feather THg concentrations in both adults at wintering (1.23 ± 1.38 mg/kg; median: 0.86 mg/kg, range: 0.26 to 7.67 mg/kg, n = 27) and juveniles at natal grounds (0.99 ± 1.02 mg/kg; median: 0.57 mg/kg; range: 0.22 to 4.40 mg/kg, n = 33). Overall, 52 (86.67%) of the 60 sampled individuals fell in the no apparent effect risk category, while 7 (11.67%) and 1 (1.67%) were in the low risk and moderate risk categories, respectively. No current studies are found in other locations worldwide.

Greater Sand Plover. This species is currently listed as Least Concern, though an apparent decline has been observed[1]. The feather THg concentrations in juveniles sampled from Broome and Tiaozini likely reflected the Hg load at Mongolia inland in a low temperate zone. The non-breeding grounds here are mainly in South China and a large stretch range of south-east Asia and Australia. The feather THg concentration in adults at non-breeding grounds (0.97 ± 0.59 mg/kg; median: 0.94 mg/kg; range: 0.25 to 2.50 mg/kg, n = 27), approximately two times higher than the feather THg concentrations in juveniles from breeding grounds (0.47 ± 0.29 mg/kg; median: 0.39 mg/kg, range: 0.21 to 1.41 mg/kg; n = 18; $F_{1,43}$ = 14.21, p = 0.00049). Overall, 42

(93.33%) of the 45 samples were in no apparent effect risk category, while 3 (6.66 %) fell in a low-risk category. To the best of our knowledge, the Hg data from other regions were not available.

Bar-tailed Godwit. This species is a remarkable long-distance migrant and is currently considered Near Threatened according to IUCN Red List criteria with a declining population trend[1]. We sampled 30 juveniles thought to originate from their birthplace in the high-Arctic region, including mainly Russian Siberia (n = 27) and Alaska (n = 3). Adults captured at the wintering site (Roebuck Bay and 80 Mile Beach, n = 9) and the over-summering site (Western Port, n = 2) and stopovers at Yalujiang (n = 2) and Yangkou (n = 1) were thought to replace their feathers at non-breeding grounds of north-west Australia and uncertain locations of south-east Asia or Australia, respectively. The adults grown their feathers at wintering sites had a mean feather THg concentration of 1.39 ± 0.35 mg/kg (median: 1.33 mg/kg; range: 0.71 to 2.07 mg/kg, n = 14), approximately three times higher compared to Hg exposure in juveniles with feathers grown at breeding grounds (0.63 ± 0.81 mg/kg; median: 1.33 mg/kg; range: 0.30 to 4.85 mg/kg, n = 30, $F_{1, 42} = 42.60$, $p < 0.000001$). Overall, 41 (91.11%) of 45 samples were within the no apparent effect risk category, while 3 (6.67%) and 1 (2.22%) were within the low risk and moderate risk categories, respectively.

Supplementary Table 1. Feather total Hg concentrations (mg/kg, dry weight) among shorebird species along the EAAF. The order of species was based on the median value.

Species	n	mean	sd	min	median	max	percentile25	percentile75
Marsh Sandpiper	63	4.39	5.35	0.35	3.40	38.63	1.81	4.78
Pied Avocet	7	2.94	2.44	0.30	2.85	7.60	1.35	3.57
Spotted Redshank	1	2.85	NA	2.85	2.85	2.85	2.85	2.85
Temminck's Stint	3	2.75	1.77	1.00	2.71	4.53	1.86	3.62
Black-tailed Godwit	1	2.67	NA	2.67	2.67	2.67	2.67	2.67
Common Redshank	63	3.65	3.34	0.50	2.44	15.94	1.71	4.03
Kentish Plover	36	2.78	3.11	0.17	2.01	17.83	1.49	3.06
Common Greenshank	9	2.31	1.51	0.70	1.90	5.14	1.28	2.46
Sharp-tailed Sandpiper	32	2.32	1.76	0.68	1.72	8.33	1.11	2.84
Greater Painted-snipe	1	1.64	NA	1.64	1.64	1.64	1.64	1.64
Spoonbilled Sandpiper	3	1.86	1.00	1.00	1.63	2.96	1.32	2.30
Dunlin	148	2.10	1.71	0.29	1.58	11.36	1.05	2.69
Great Knot	42	1.76	1.05	0.11	1.55	5.33	1.22	2.04
Wood Sandpiper	1	1.48	NA	1.48	1.48	1.48	1.48	1.48
Terek Sandpiper	65	1.50	0.96	0.28	1.30	4.08	0.78	2.04
Grey Plover	70	1.27	0.63	0.34	1.22	4.36	0.85	1.54
Ruddy Turnstone	49	1.27	0.56	0.52	1.19	3.16	0.85	1.53
Sanderling	44	1.53	1.27	0.35	1.05	6.27	0.55	2.16
Red Knot	45	1.30	1.04	0.31	1.00	6.28	0.77	1.41
Lesser Sand Plover	21	1.12	0.67	0.41	0.96	3.25	0.71	1.15
Curlew Sandpiper	66	1.54	1.86	0.32	0.95	13.13	0.68	1.73
Common Snipe	15	1.81	2.44	0.31	0.94	8.66	0.47	1.59

Black-winged stilt	1	0.79	NA	0.79	0.79	0.79	0.79	0.79
Asian Dowitcher	1	0.76	NA	0.76	0.76	0.76	0.76	0.76
Red-necked Stint	60	1.10	1.19	0.22	0.71	7.67	0.52	1.23
Grey-tailed Tattler	20	1.04	0.95	0.28	0.69	4.14	0.46	1.58
Common Sandpiper	3	0.68	0.20	0.49	0.67	0.89	0.58	0.78
Eurasian Whimbrel	16	0.69	0.28	0.33	0.67	1.26	0.46	0.85
Pacific Golden Plover	4	0.72	0.48	0.29	0.61	1.39	0.44	0.89
Bar-tailed Godwit	45	0.87	0.77	0.30	0.60	4.85	0.40	1.26
Greater Sand Plover	45	0.77	0.55	0.21	0.60	2.50	0.36	1.08
Broad-billed Sandpiper	2	0.56	0.33	0.33	0.56	0.79	0.45	0.68
Long-toed Stint	2	0.51	0.28	0.31	0.51	0.70	0.41	0.60

Supplementary Table 2. Variable importance ordered by importance and a number of linear mixed-effects models with all combinations of predictor variables included (4 candidate models, ΔAICc ≤ 2). Habitat Preference: each species' typical dependency on coastal habitats during the non-breeding season (NCO: non-coastal obligate, <100% use of coastal habitats; CO: coastal obligate, 100% use). Foraging Stratum: the prevalence of foraging time on or just below the water surface, indicated by the estimated use percentage (surface: <50%; depth: 50%−100%). Diet: the percentage of consumed invertebrates (A: 50%−79%; B: 80%−100%).

	**Region**	**Habitat Preference**	**Diet**	**Foraging Stratum**
**Sum of weights:**	1	0.32	0.17	0.15
**N containing models:**	4	1	1	1

Supplementary Table 3. The pairwise comparisons for region variable in the full model (logHg ~ Foraging Stratum + Region + Habitat Preference + Diet + (1| Species) + (1| Year)).

Emmeans

Region	emmean	SE	df	lower.CL	upper.CL
Arctic	−0.03	0.05	57.10	−0.13	0.08
Australia	0.13	0.05	54.20	0.03	0.23
South China	0.21	0.06	73.10	0.09	0.33
Temperate	0.02	0.06	68.10	−0.09	0.13
Yellow Sea	0.10	0.08	168.40	−0.05	0.25

Results are averaged over the levels of: Foraging Stratum, Habitat Preference, Diet
Degrees-of-freedom method: Kenward-roger
Confidence level used: 0.95

Contrasts

contrast	estimate	SE	df	t.ratio	p.value
Arctic-Australia	−0.16	0.03	468.00	−4.77	<.0001
Arctic-South China	−0.24	0.05	601.00	−4.73	<.0001
Arctic-Temperate	−0.05	0.05	545.00	−0.98	0.86
Arctic-Yellow Sea	−0.13	0.08	489.00	−1.70	0.44
Australia-South China	−0.08	0.05	260.00	−1.51	0.55
Australia-Temperate	0.11	0.05	389.00	2.29	0.15
Australia-Yellow Sea	0.03	0.08	450.00	0.42	0.99
South China-Temperate	0.19	0.04	746.00	4.61	<.0001
South China-Yellow Sea	0.11	0.07	628.00	1.52	0.55
Temperate-Yellow Sea	−0.08	0.07	650.00	−1.18	0.77

Results are averaged over the levels of: Foraging Stratum, Habitat Preference, Diet
Degrees-of-freedom method: Kenward-roger
p-value adjustment: tukey method for comparing a family of 5 estimates

Supplementary Table 4 The summarized table for samples by region, species and age class.

**Region**	**Number of species**	**Adult**	**Juvenile**	**Unknown age**	**Total number**
Arctic	13	0	211	0	211
Temperate	14	16	151	1	168
Temperate-Arctic	1[a]	62	81	0	143
Yellow Sea	6	27	0	0	27
South China	14	115	1	0	116
Australia	13	220	0	0	220
South-east Asia, Australia	10	61	1	0	62
Unknown region	12	23	5	9	37
Total	33[b]	378	363	1	984

a: The species used the Temperate-Arctic region is Dunlin. b: Some species were collected across regions, thus the total number of species does not equal the sum of the number of species sampled in individual region.

Supplementary Table 5. Multicollinearity (indicated by VIF) in full model logHg ~ Foraging Stratum + Region + Habitat Preference+ Diet + (1| Species) + (1| Year). A VIF less than 5 indicates a low correlation of that predictor with other predictors. A value between 5 and 10 indicates a moderate correlation, while VIF values larger than 10 indicate a high, not tolerable correlation of model predictors.

Term	VIF	VIF 95% CI	Increased SE	Tolerance	Tolerance 95% CI
Foraging Stratum	1.04	[1.00, 1.30]	1.02	0.97	[0.77, 1.00]
Region	1.07	[1.03, 1.22]	1.03	0.94	[0.82, 0.98]
Habitat Preference	1.13	[1.06, 1.26]	1.06	0.89	[0.79, 0.94]
Diet	1.11	[1.05, 1.24]	1.05	0.90	[0.80, 0.95]

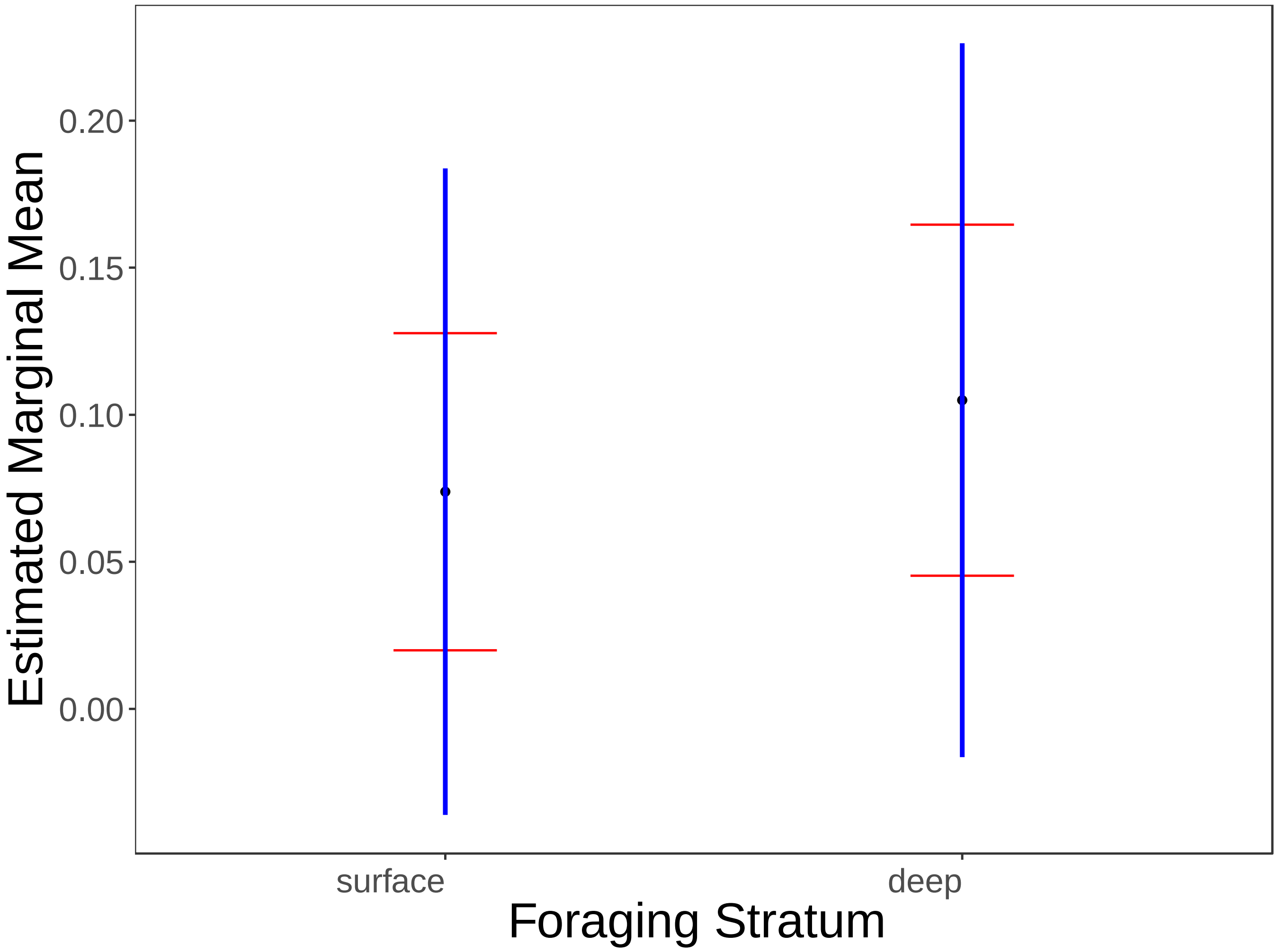

Supplementary Figure 1. Graphic display of the estimated means, standard errors (red horizontal bars) and 95% confidence intervals (vertical blue lines) for foraging stratum on the full model (logHg ~ Foraging Stratum + Region + Habitat Preference+ Diet + (1| Species) + (1| Year)). Foraging Stratum: the prevalence of foraging time on or just below the water surface, indicated by the estimated use percentage (surface: <50%; depth: 50-100%).

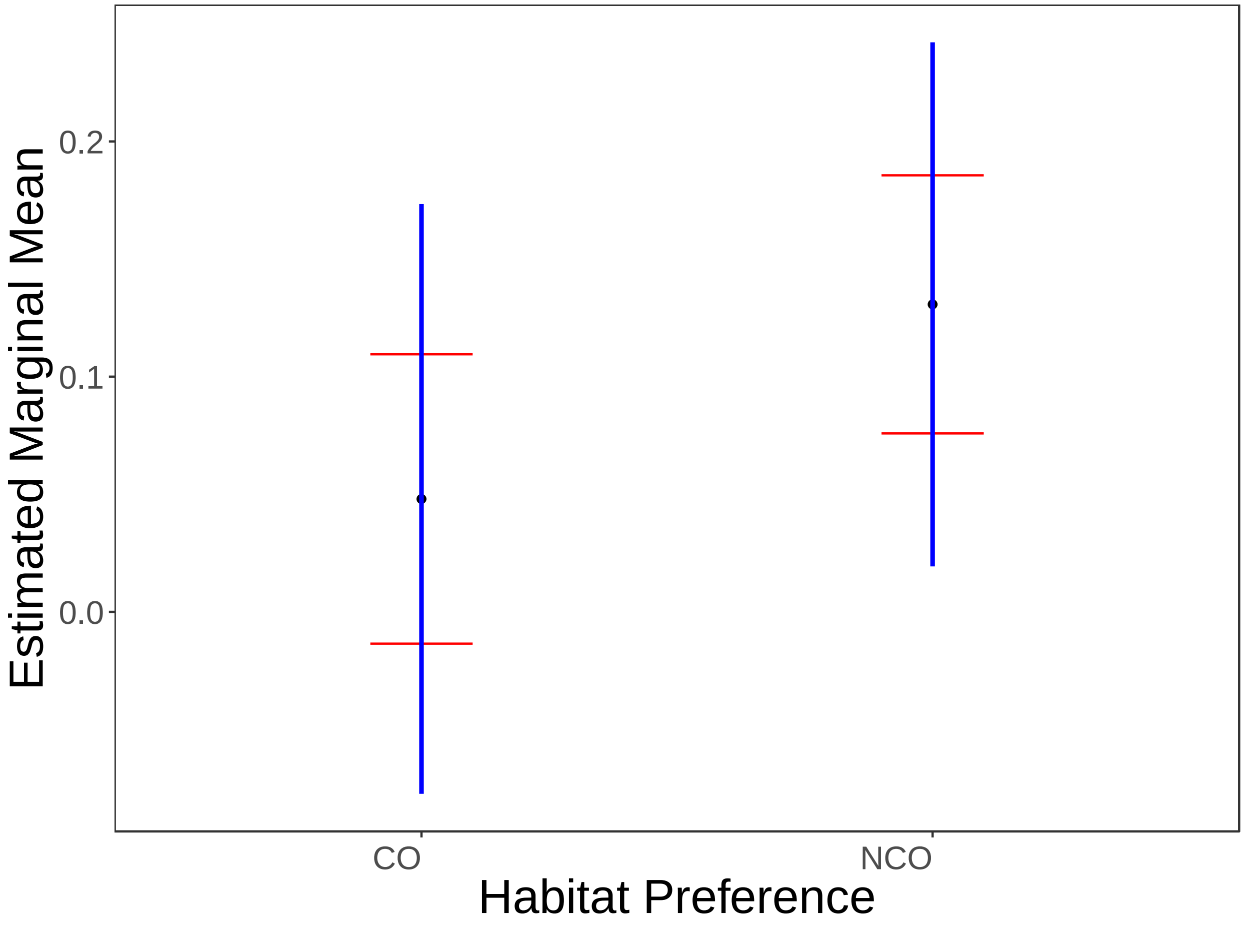

Supplementary Figure 2. Graphic display of the estimated means, standard errors (red horizontal bars) and 95% confidence intervals (vertical blue lines) for habitat preference on the full model (logHg ~ Foraging Stratum + Region + Habitat Preference+ Diet + (1| Species) + (1| Year)). Habitat Preference: each species' typical dependency on coastal habitats during the non-breeding season (CO: coastal obligate, 100% use of coastal habitats; NCO: non-coastal obligate, <100% use of coastal habitats).

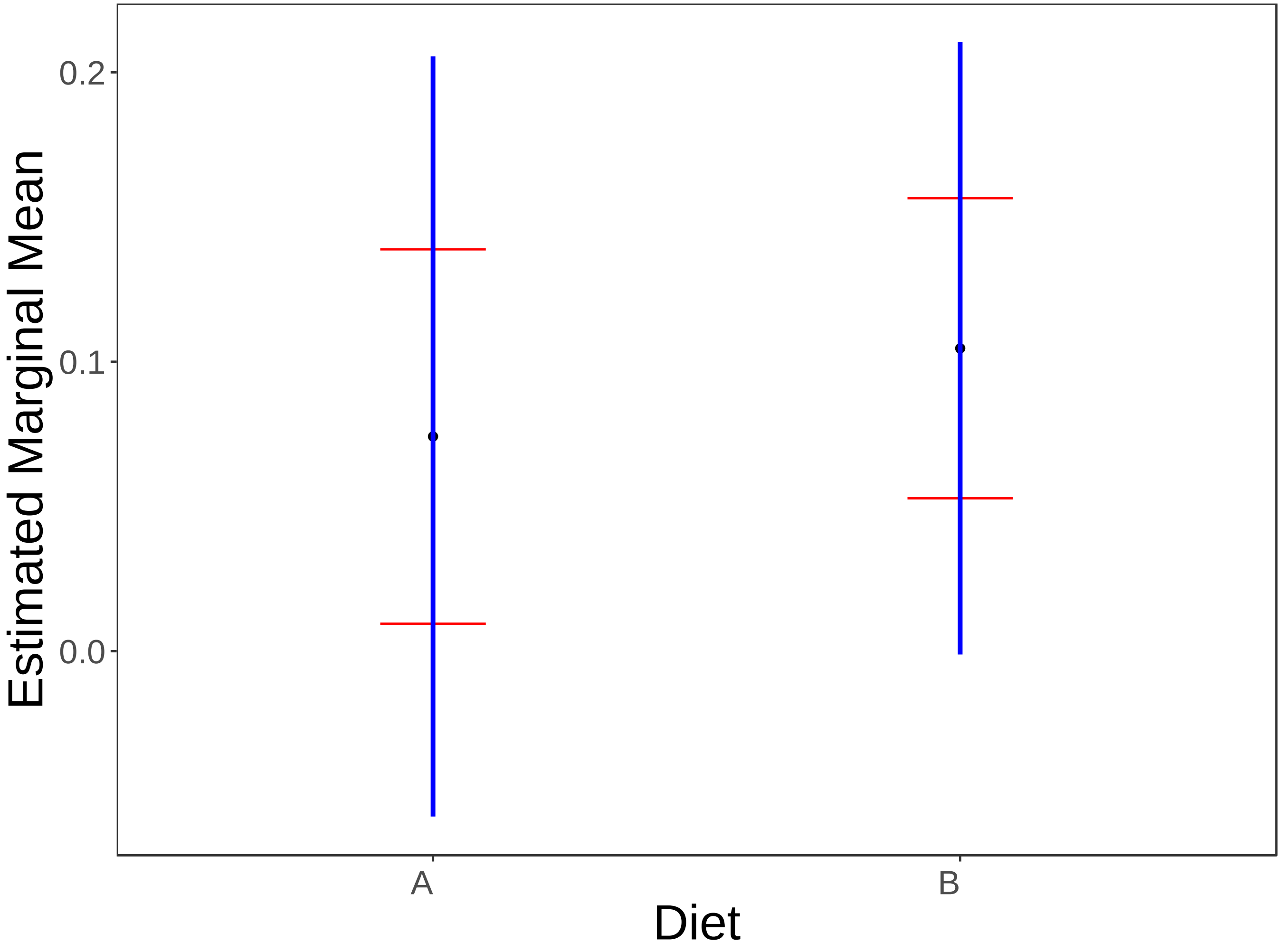

Supplementary Figure 3. Graphic display of the estimated means, standard errors (red horizontal bars) and 95% confidence intervals (vertical blue lines) for diet on the full model (logHg ~ Foraging Stratum + Region + Habitat Preference+ Diet + (1| Species) + (1| Year)). Diet: the percentage of consumed invertebrates (A: 50%−79%; B: 80%−100%).

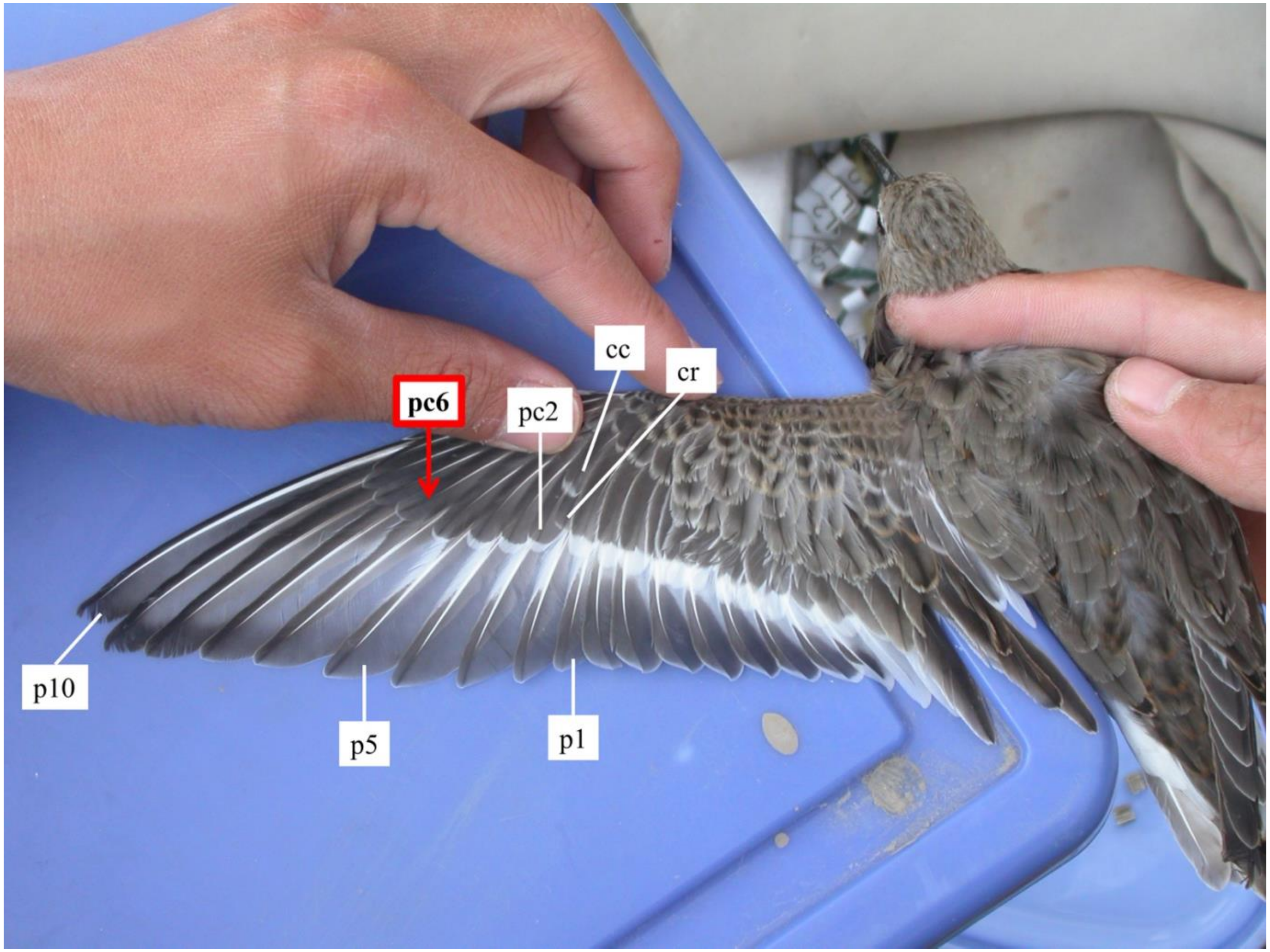

Supplementary Figure 4. The position of primary coverts in a common shorebird species: Dunlin[7]. The feather labelled as pc6 is the sixth primary covert, our target feather for this exercise. If pc6 was unavailable, the nearest, most recently completely-grown primary covert, which usually has similar concentrations, was used. Some of the primaries (p), the carpal covert (cc) and carpal remex (cr) are also shown in this figure.

Supplementary References

1. IUCN. The IUCN Red List of Threatened Species. Version 2022-2. www.iucnredlist.org (2023).
2. Burger, J., Seyboldt, S., Morganstein, N. & Clark, K. Heavy metals and selenium in feathers of three shorebird species from Delaware bay. *Environ. Monit. Assess.* **28**, 189–198 (1993).
3. Burger, J. *et al.* Heavy metals in Biota in Delaware Bay, NJ: Developing a food web approach to contaminants. *Toxics* **7**, 1–16 (2019).
4. Tsipoura, N. *et al.* Metal Levels in Shorebird Feathers and Blood During Migration Through Delaware Bay. *Arch. Environ. Contam. Toxicol.* **72**, 562–574 (2017).
5. Burger, J. *et al.* Mercury, lead, cadmium, arsenic, chromium and selenium in feathers of shorebirds during migrating through Delaware Bay, New Jersey: Comparing the 1990s and 2011/2012. *Toxics* **3**, 63–74 (2015).
6. Lucia, M., Bocher, P., Cosson, R. P., Churlaud, C. & Bustamante, P. Evidence of species-specific detoxification processes for trace elements in shorebirds. *Ecotoxicology* **21**, 2349–2362 (2012).
7. Choi, C., Hua, N., Persson, C., Chiang, C. & Ma, Z. Age-related plumage differences of Dunlins along the East Asian-Australasian Flyway. *J. F. Ornithol.* **81**, 99–111 (2010).
