# Supplementary material for: Mercury contamination is an invisible threat to declining migratory shorebirds along the East Asian-Australasian Flyway
Source: Commun Biol. 2024 May 16;7:585. doi: 10.1038/s42003-024-06254-x (PMC11098816; doi:10.1038/s42003-024-06254-x)
Supplement: Supplementary file 2 — Description of Additional Supplementary Files [file 42003_2024_6254_MOESM2_ESM.pdf]

Description of Additional Supplementary Files

File name: Supplementary Data 1
Description: The source data analyses and behind the figures in this study.

File name: Supplementary Data 2
Description: Study species, age classes, captured sites, captured years and month captured countries, indicated regions and sample sizes for this study.
