# Supplementary material for: Mercury contamination is an invisible threat to declining migratory shorebirds along the East Asian-Australasian Flyway
Source: Commun Biol. 2024 May 16;7:585. doi: 10.1038/s42003-024-06254-x (PMC11098816; doi:10.1038/s42003-024-06254-x)
Supplement: Supplementary file 5 — Reporting Summary [file 42003_2024_6254_MOESM5_ESM.pdf]

nature portfolio

Corresponding author(s): Luke Gibson and Chi-Yeung Choi

Last updated by author(s): Mar 24, 2024

Reporting Summary

Nature Portfolio wishes to improve the reproducibility of the work that we publish. This form provides structure for consistency and transparency in reporting. For further information on Nature Portfolio policies, see our Editorial Policies and the Editorial Policy Checklist.

Statistics

For all statistical analyses, confirm that the following items are present in the figure legend, table legend, main text, or Methods section.

n/a	Confirmed	
☐	☒	The exact sample size (*n*) for each experimental group/condition, given as a discrete number and unit of measurement
☐	☒	A statement on whether measurements were taken from distinct samples or whether the same sample was measured repeatedly
☐	☒	The statistical test(s) used AND whether they are one- or two-sided<br>*Only common tests should be described solely by name; describe more complex techniques in the Methods section.*
☐	☒	A description of all covariates tested
☐	☒	A description of any assumptions or corrections, such as tests of normality and adjustment for multiple comparisons
☐	☒	A full description of the statistical parameters including central tendency (e.g. means) or other basic estimates (e.g. regression coefficient) AND variation (e.g. standard deviation) or associated estimates of uncertainty (e.g. confidence intervals)
☐	☒	For null hypothesis testing, the test statistic (e.g. *F*, *t*, *r*) with confidence intervals, effect sizes, degrees of freedom and *P* value noted<br>*Give P values as exact values whenever suitable.*
☒	☐	For Bayesian analysis, information on the choice of priors and Markov chain Monte Carlo settings
☒	☐	For hierarchical and complex designs, identification of the appropriate level for tests and full reporting of outcomes
☒	☐	Estimates of effect sizes (e.g. Cohen's *d*, Pearson's *r*), indicating how they were calculated

Our web collection on statistics for biologists contains articles on many of the points above.

Software and code

Policy information about availability of computer code

Data collection	We combined knowledge of moult strategy and life history with a standardised sampling protocol and collected shorebird flight feathers.
Data analysis	Total mercury concentrations were log10 transformed to meet the assumption of normality. All analyses were performed in R statistical software, using packages “performance” (check_collinearity function, version 0.10.8), “ggplot2” , “lme4” (lmer function, version 1.1-29), “MuMIn” (model.sel function, version 1.46.0 and “emmeans” (model.avg function, version 1.8.2). Values were shown as mean ± s.d. (median; range, sample size) and estimates from the LMMs were shown as mean and 95% confidence intervals (CI).

For manuscripts utilizing custom algorithms or software that are central to the research but not yet described in published literature, software must be made available to editors and reviewers. We strongly encourage code deposition in a community repository (e.g. GitHub). See the Nature Portfolio guidelines for submitting code & software for further information.

Data

Policy information about availability of data

All manuscripts must include a data availability statement. This statement should provide the following information, where applicable:

- Accession codes, unique identifiers, or web links for publicly available datasets
- A description of any restrictions on data availability
- For clinical datasets or third party data, please ensure that the statement adheres to our policy

All data that support the findings of this study are included within this paper and its Supplementary Information files

Research involving human participants, their data, or biological material

Policy information about studies with human participants or human data. See also policy information about sex, gender (identity/presentation), and sexual orientation and race, ethnicity and racism.

Reporting on sex and gender	No applicable.
Reporting on race, ethnicity, or other socially relevant groupings	No applicable.
Population characteristics	No applicable.
Recruitment	No applicable.
Ethics oversight	No applicable.

Note that full information on the approval of the study protocol must also be provided in the manuscript.

Field-specific reporting

Please select the one below that is the best fit for your research. If you are not sure, read the appropriate sections before making your selection.

☐ Life sciences ☐ Behavioural & social sciences ☒ Ecological, evolutionary & environmental sciences

For a reference copy of the document with all sections, see nature.com/documents/nr-reporting-summary-flat.pdf

Ecological, evolutionary & environmental sciences study design

All studies must disclose on these points even when the disclosure is negative.

Study description	We assessed mercury (Hg) contamination in 984 individuals across 33 migratory shorebird species along the East Asian-Australasian Flyway.
Research sample	Feather samples were collected from 18 sites in China and Australia. Target species were selected based on the distribution of coasts in China and Australia, moult ecology, and ability to catch them. The specific flight feathers (PC6) from juveniles indicate Hg concentrations at breeding/natal grounds, while from adults represent Hg contamination at non-breeding grounds.
Sampling strategy	Feathers were stored in paper envelopes or plastic zip-loc bags at room temperature.
Data collection	Target species were selected based on the distribution of coasts in China and Australia, moult ecology, and ability to catch them.
Timing and spatial scale	Along the Chinese coast, migratory shorebirds (n = 622) were captured using mist nets or clap nets and sampled during non-breeding periods (from January to April and August to December) between 2019 and 2022.In Australia, birds (n = 362) were captured by canon nets between 2004 and 2016.
Data exclusions	We excluded 108 individuals with either unclear moult areas or broad moult origins for analysis of predicting factors in Hg contamination.
Reproducibility	Relative percentage differences (mean ± sd) for duplicate samples were less than 15%.
Randomization	We tried to spread the sampling effort evenly across the season (i.e., avoid collecting all the feather samples during one occasion).
Blinding	Blinding is not relevant to our study due to strict QAQC procedure.

Did the study involve field work? ☒ Yes ☐ No

Field work, collection and transport

Field conditions	For details of capture time period, locations and collected periods, see Table S4
Location	For details of capture locations and collected periods, see Table S4
Access & import/export	We strictly followed the laws to import feathers from Australia to China.

Disturbance	not applicable

Reporting for specific materials, systems and methods

We require information from authors about some types of materials, experimental systems and methods used in many studies. Here, indicate whether each material, system or method listed is relevant to your study. If you are not sure if a list item applies to your research, read the appropriate section before selecting a response.

Materials & experimental systems

n/a	Involved in the study
☒	☐ Antibodies
☒	☐ Eukaryotic cell lines
☒	☐ Palaeontology and archaeology
☐	☒ Animals and other organisms
☒	☐ Clinical data
☒	☐ Dual use research of concern
☒	☐ Plants

Methods

n/a	Involved in the study
☒	☐ ChIP-seq
☒	☐ Flow cytometry
☒	☐ MRI-based neuroimaging

Animals and other research organisms

Policy information about studies involving animals; ARRIVE guidelines recommended for reporting animal research, and Sex and Gender in Research

Laboratory animals	No applicable.
Wild animals	Along the Chinese and Australian coasts, migratory / wintering shorebirds were captured using mist nets or clap nets and sampled feather immediately, then released. For details of capture species, sampling period, locations and collected periods, see Table S4
Reporting on sex	The sex information of species were not intentionally collected for all individuals.
Field-collected samples	Feather samples in this research were collected immediately when birds caught.
Ethics oversight	All experimental protocols of bird capture and handling were strictly complied with the requirement of Chinese Wild Animal Protection Law and approved by Liaoning Provincial Forestry and Grassland Bureau (2020-53; 2022-24); Leizhou Natural Resources Bureau (2019-48), Yauljiang National Nature Reserve, Yancheng National Nature Reserve, Chongming Dongtan National Nature Reserve and Zhanjiang National Nature Reserve for samples collected in Yalujiang, Tiaozini, Yangkou, Chongming Dongtan and Leizhou and by the Agriculture, Fisheries and Conservation Department of the Government of the Hong Kong SAR, China for samples collected at MaiPo Nature Reserve. Samples collection in Australia was carried out under the license of the Australian Bird and Bat Banding Scheme (ABBBS). The catching and collection of samples on the shores of Roebuck Bay were conducted with the permission of the Yawuru People. The samples collected in Northwest Australia were under the Regulation 17 Licence 08-00741-3 authorised to CJH from the Department of Biodiversity, Conservation and Attractions. The sample collected in Southeast Australia under the license CVL1337 issued to JTC by the Department of National Parks, Sport and Racing, Queensland Government and Department of Primary Industries Animal Ethics license CA 2015-03-845. The samples from Victoria, Australia, were collected under Phillip Island Nature Parks Animal Ethics Committee Permits; 4.2004, 4.2007.

Note that full information on the approval of the study protocol must also be provided in the manuscript.
